# *Brinp1*^*−/−*^ mice exhibit autism-like behaviour, altered memory, hyperactivity and increased parvalbumin-positive cortical interneuron density

**DOI:** 10.1186/s13229-016-0079-7

**Published:** 2016-03-31

**Authors:** Susan R. Berkowicz, Travis J. Featherby, Zhengdong Qu, Aminah Giousoh, Natalie A. Borg, Julian I. Heng, James C. Whisstock, Phillip I. Bird

**Affiliations:** Department of Biochemistry and Molecular Biology, Monash University, Clayton, VIC 3800 Australia; Australian Regenerative Medicine Institute, Monash University, Clayton, VIC 3800 Australia; Florey Institute of Neuroscience and Mental Health, Parkville, VIC 3052 Australia; Australian Research Council Centre of Excellence in Advanced Molecular Imaging, Monash University, Clayton, VIC 3800 Australia

**Keywords:** *Brinp1*, Knock-out, Cortex, Parvalbumin, Interneuron, Neurodevelopment, Autism spectrum disorder, Hyperactivity

## Abstract

**Background:**

BMP/RA-inducible neural-specific protein 1 (*Brinp1*) is highly conserved in vertebrates, and continuously expressed in the neocortex, hippocampus, olfactory bulb and cerebellum from mid-embryonic development through to adulthood.

**Methods:**

*Brinp1* knock-out (*Brinp1*^−/−^) mice were generated by Cre-recombinase-mediated removal of the third exon of *Brinp1*. Knock-out mice were characterised by behavioural phenotyping, immunohistochemistry and expression analysis of the developing and adult brain.

**Results:**

Absence of *Brinp1* during development results in a behavioural phenotype resembling autism spectrum disorder (ASD), in which knock-out mice show reduced sociability and changes in vocalisation capacity. In addition, *Brinp1*^−/−^ mice exhibit hyper-locomotor activity, have impaired short-term memory, and exhibit poor reproductive success.

*Brinp1*^−/−^ mice show increased density of parvalbumin-expressing interneurons in the adult mouse brain. *Brinp1*^−/−^ mice do not show signs of altered neural precursor proliferation or increased apoptosis during late embryonic brain development. The expression of the related neuronal migration genes *Astn1* and *Astn2* is increased in the brains of *Brinp1*^−/−^ mice, suggesting that they may ameliorate the effects of *Brinp1* loss.

**Conclusions:**

*Brinp1* plays an important role in normal brain development and function by influencing neuronal distribution within the cortex. The increased cortical PV-positive interneuron density and altered behaviour of *Brinp1*^−/−^ mice resemble features of a subset of human neurological disorders; namely autism spectrum disorder (ASD) and the hyperactivity aspect of attention deficit hyperactivity disorder (ADHD).

**Electronic supplementary material:**

The online version of this article (doi:10.1186/s13229-016-0079-7) contains supplementary material, which is available to authorized users.

## Background

Many neurodevelopmental disorders (NDDs) result from rare genetic alterations including copy number variations (CNVs), single nucleotide polymorphisms (SNPs) and de novo mutations in genes affecting a variety of cellular processes [1–3]. Autism spectrum disorder (ASD) is a NDD that affects an estimated 0.6 % of children [4, 5], with similar prevalence in adults [6]. Clinical diagnosis is based on atypical social behaviour, impairments in verbal and non-verbal communication, and patterns of restricted interests and repetitive behaviours [7]. Autistic patients commonly have moderate to severe impairments in expressive language [8, 9]. ASD also frequently presents with at least one other NDD, for example attention deficit hyperactivity disorder (ADHD), anxiety disorder, or intellectual disability [10, 11]. Notably, approximately 28 % of patients diagnosed with ASD also present with ADHD, diagnosed in childhood by inattention, hyperactivity and impulsivity [7, 12]. Whist the underlying pathology of such NDDs is not fully understood, interneuron abnormalities have shown to be a key contributor to ASD, and other NDD pathologies [13–17].

Members of the Membrane Attack Complex/Perforin (MACPF) protein superfamily share a domain that facilitates oligomerisation and membrane association [18, 19]. BMP/RA-inducible neural-specific protein 1 (BRINP1) is one of five MACPF members expressed predominantly in the nervous system. The 85-kDa BRINP1 protein is over 50 % identical to two other MACPF proteins, BRINP2 and BRINP3 [20], and all three are expressed in an overlapping pattern in neurons in the developing and mature brain.

The physiological role of BRINP1 is not understood. It has been suggested to function in cell cycle regulation [20, 21]. Kobayashi et al. recently produced mice carrying a homozygous exon-8 deletion in *Brinp1.* Adult mutant mice display increased neuronal proliferation in the hippocampus and more parvalbumin-positive interneurons in the CA1 region of the hippocampus. The mice also show behavioural alterations suggested to be relevant to schizophrenia or ADHD [22]. *Brinp1* has also been implicated with neural plasticity of song learning in birds [23, 24] and is reported to be lost in multiple cancers [25–27]. The subcellular localisation of *Brinp1* is unknown, but EGFP-tagged *Brinp1* has been observed in the endoplasmic reticulum of the PC12 (rat) cell line, suggesting it functions in the secretory/endolysosomal system or is released from cells [20].

**Fig. 1:**
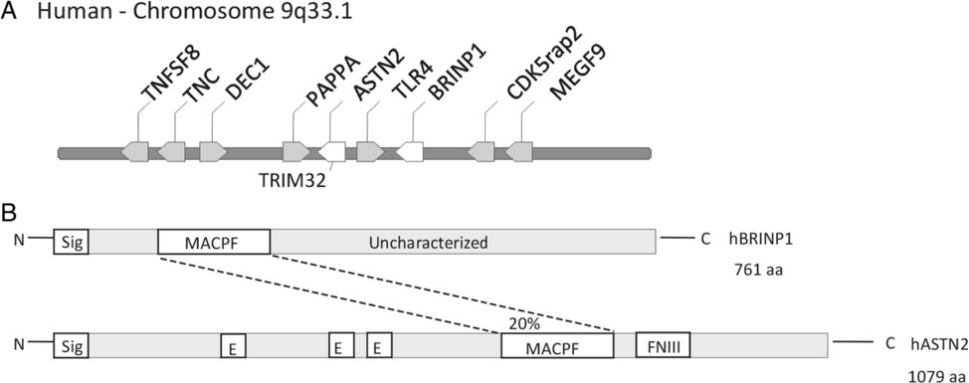
*BRINP1* and *ASTN2* share homology and a common locus. **a** Schematic of the *BRINP1/ASTN2* locus at 9q33.1. **b** BRINP1 and ASTN2 show 20 % homology via a common MACPF domain. Sig = Signal sequence, E = EGF-like domain, FNIII = Fibronectin III domain

*BRINPs* share homology with Astrotactin 1 (*ASTN1*) and Astrotactin 2 (*ASTN2*); two neural proteins that facilitate glial-guided neuronal migration [28, 29]. In humans, *BRINP1* and *ASTN2* are situated at a common chromosome locus: 9q33.1 (Fig. 1), whilst *BRINP2*, *BRINP3* and *ASTN1* are linked on chromosome 1. Genome-wide association studies (GWAS) have found *BRINP1* to be associated with neurological disorders: *BRINP1* (*DBC1*) variation is reported as a potential biomarker that discriminates with first episode schizophrenia [30]; *BRINP1* SNPs show association with Parkinson’s disease [31, 32], as well as with late-onset dementia [33]. *ASTN2* has been identified as a risk gene in ASD, ADHD and schizophrenia, with copy number variations (CNVs) of the gene detected in patients [34–37]. Recently, Lionel et al. reported 58 CNVs at the 9q33.1 loci associated with a NDD diagnosis. Forty-six sequenced CNVs involved *ASTN2*/*TRIM32*, whilst seven extended to *BRINP1* [35]. Such findings suggest that alterations to BRINP1 function may also contribute to NDD.

To further investigate the role of *BRINP1* in brain development and cognitive function, we generated conditional exon 3-deleted *Brinp1*^*−/−*^ mice, via the LoxP/Cre-recombinase system. We demonstrate that these mice exhibit reduced reproductive fecundity, altered parvalbumin interneuron density in both the neocortex and hippocampus, with no change in cell proliferation in the developing embryonic brain (E18.5). In addition, the mice exhibit a social communication phenotype reminiscent of behavioural traits seen in human autism spectrum disorder.

## Methods

### Gene targeting

A targeting vector was constructed to alter the *Brinp1* locus in mouse embryonic stem (ES) cells by homologous recombination following the general strategy outlined by Teoh et al., 2014 [38]. The vector was built using bacterial artificial chromosome (BAC) clone RP23-85B13 as a source of *Brinp1* DNA. This vector comprised a neomycin transcriptional unit flanked by flippase (Flp) recognition target (FRT) elements placed in intron 3. A loxP element was placed in the same intron immediately downstream of the neomycin cassette, whilst an upstream loxP element was placed in intron 2. Cre-recombinase-mediated deletion of exon 3 was designed to generate a frame shift, resulting in a stop codon in the fourth exon of *Brinp1*. The targeting construct was electroporated into Bruce 4 C57BL/6-derived embryonic stem (ES) cells, and the targeted clone carrying the targeted allele (*Brinp1*^*tm1Pib*^ (MGI: 5604540)) was identified by Southern analysis. A correctly targeted clone was injected into BALB/c blastocysts to generate chimeric mice, which were crossed to C57BL/6 Cre deleter transgenic mice Tg(CMV-cre)1Cgn to remove exon 3 and the neomycin cassette from the targeted allele. This produced animals carrying the *Brinp1*^*tm1.1Pib*^ mutation (MGI: 5604542). In parallel, chimeric mice were crossed to C57BL/6 Flp deleter transgenic mice to remove the neomycin cassette only (*Brinp1*^*tm1.2Pib*^ (MGI: 5604543)). ‘Floxed’ mice heterozygous for the *Brinp1*^*tm1.1Pib*^ mutation were inter-crossed to generate mice of all three genotypes: *Brinp1*^*+/+*^ (wild type, WT); *Brinp1*^*+/tm1.1Pib*^ (het); and *Brinp1*^*tm1.1Pib/tm1.1Pib*^ (*Brinp1*^*−/−*^).

### Genomic analysis

The WT, *Brinp1*-targeted, and *Brinp1*-floxed mouse lines were all verified by Southern analysis. Genomic DNA isolated from the spleen was digested with *Pst* I and probed with a 500 bp 5′ homology probe. A 3′ homology arm probe was used for blotting of genomic DNA digested with *Bgl* III. An internal *Bgl* III probe was used to rule out random integration into the genome. PCR analysis confirmed absence of the neomycin cassette in floxed animals.

### Immunoblotting

Whole brain lysates were made from *Brinp1*^−/−^ and WT mice at postnatal day 12. Samples were run on a 10 % SDS-polyacrylamide gel and transferred to nitrocellulose membrane. The blots were probed with a custom-made rat polyclonal antibody to human BRINP1 (1:200). For BRINP1 antibody production, rats were immunised with gel slices containing purified, denatured recombinant BRINP1 produced in a *Baculovirus* expression system. Antiserum was validated by indirect immunofluorescence of COS-1 cells transiently expressing human BRINP1 and immunoblotting of corresponding COS-1 cell lysates, following approaches described by Teoh et al. [39]. No signal was detected in mock-transfected (control) COS-1 cell samples. The secondary antibody was an anti-Rat HRP (Rockland, 1:5000); loading control: βIII-tubulin (Covance, 1:1000).

### RT-PCR

RNA was extracted from the whole brain of WT and *Brinp1*^−/−^ embryos (E18.5) and reverse transcribed into complementary DNA (cDNA) (SSIII First-Strand Synthesis, Life Sciences). Primers were designed to exon 2: 5′-CTGGGACAGACCAACATGTCTC and exon 6: 3′-GCTCTCCGTGCTTTGCAGAAGG, to produce a 526 bp WT product or a 336-bp floxed product. PCR conditions are as follows: 95 °C 60 s (95 °C 30 s, 61 °C 30 s, 72 °C 30 s) × 35, 72 °C 120 s. WT and *Brinp1*^*−/−*^ products were cut out of a 2 % agarose gel and sequenced.

### qPCR

Primers were designed to produce a single PCR product within a range of 80–190 bp (optimal size of 150 bp). Primers were first validated by RT-PCR, checking for a single PCR product of the predicted size. PCR reactions were set up in a 96-well plate format as 10 μl reactions: 5 μl SYBR Green (Sigma), 4.1 μl of water, 0.2 μl primer 1, 0.2 μl primer 2, and 0.5 μl cDNA. Reactions were run on a Roche Light Cycler 96: 95 °C 60 s, (95 °C 30 s, 61 °C 30 s) x 45, 72 °C 120 s. Reference genes glyceraldehyde 3-phosphate dehydrogenase (GAPDH) and β-actin were used for normalisation. Results are represented as the fold change relative to WT and were analysed using unpaired Student’s *t* tests.

### Animals

C57BL/6 *Brinp1*^−/−^ animals and wild-type littermates were generated from heterozygous breeders in all studies, except for monitoring of homozygous matings. Mice were genotyped from tail snips collected at postnatal day 10 (P10). Mice were kept under standard housing conditions with a 12 h light/dark cycle. Mice were housed with mixed genotype littermates; maximum of five adults per box. All breeding and experiments were approved by the Monash University Animal Ethics Committee.

### Weighing

*Brinp1*^−/−^, het, and WT littermates were weighed weekly between 3 and 12 weeks (*n* = 8 males, 8 females per genotype). Repeat measures two-way ANOVAs were performed for comparisons between WT and knock-out mice weights.

### Reproductive phenotyping

Four breeders per genotype (WT/WT, Het/Het, *Brinp1*^*−/−*^/*Brinp1*^*−/−*^ and WT ♂/*Brinp1*^*−/−*^ ♀) were set up and monitored over four months. Mice were used as breeders at 7–8 weeks of age. The number of pups was recorded at birth, 48 h post birth, and at weaning (postnatal day 10).

### Behavioural testing

Cohort sizes were 9–12 mice per genotype, aged 3–4 months. A 1:1 ratio of females/males was tested. Mice were habituated to the testing facility for 1 week then habituated to the testing room overnight. Mice were tested blind to genotype and in random order. Tests were separated by a minimum of 1 day. WT and knock-out mice were tested in the same testing sessions. Lighting conditions were 30 lux for all behavioural tests. Testing arenas were cleaned with Equinade disinfectant (lavender scent) between trials. In all instances, mice had previously been habituated to the disinfectant whilst housed at the testing facility.

### Visual placing test

Mice were lifted by the tail to a height of 15 cm and lowered onto a mesh grid within 1 s, decelerating as the grid approached. One trial was performed per mouse. The distance of the animal’s nose from the grid was measured the moment before the mouse extended its forelimbs towards it. A single trial was performed per animal.

### Rotarod

Mice were pre-trained on the Rotarod (Ugo Basil) for two initial trials at a constant speed of 4 rpm for 5 min, followed by a third trial accelerating from 4–40 rpm over 5 min. Testing was carried out the following day by 4 × 5 min accelerating trials with an inter-trial interval of 30 min.

### Three-chamber social interaction test

Adapted from methods previously described by Silverman et al, 2010: Identical rectangular wire cages were placed in equivalent positions in the left and right chambers of a 3-chamber plexiglass box (600 × 400 × 250 mm). Mice were habituated to the empty cages in the left and right chambers (trial 1) and time interacting with each cage was recorded. In trial 2, an unfamiliar C57BL/6 J WT sex-matched mouse was introduced to one of the cages, and time interacting with each cage was again recorded. In trial 3, a novel mouse was added to the empty cage, and interaction time was compared for each mouse. Each trial lasted 10 min. The mice serving as strangers were habituated to placement under the wire cage for 5 min prior to the test. Mice were tracked using CleverSys Tracking and Topscan software. The interaction zone was defined by the software as an unmarked perimeter zone of 2 cm around the metal cages. Interaction time was defined as nose within the interaction zone. The chambers were cleaned between trials with Equinade disinfectant (lavender scent).

### Ultrasonic vocalisation (USV) recording of male mice

Based on methods described by Maggio et al. [41]: A cohort of adult male mice was presented with female urine to induce ultrasonic vocalisation in a test setting. Twelve hours prior to testing of males, vaginal smears were taken from an experimentally naïve cohort of female WT littermates of a similar age. After the smear was air-dried, nuclei were visualised by a Diff-Quick stain, and the three stages of the estrus cycle were identified by light microscope. Only urine from females predicted to be in a proestrous or estrus on the day of testing were used. In order to reduce variability and ensure that mice make USVs, prior to testing, males were put in a cage with a proestrous or estrus female for 5 min. For testing, 5 min prior to the urine exposure, male mice were habituated to a cotton-tipped applicator. The applicator was then switched for a new applicator dipped in urine from estrus or proestrous female mice. USVs were recorded for 3 min using an Avisoft Recorder. Parameters were analysed using Avisoft-SASLab Pro software. Call types were designated using call classifications previously assigned by Scattoni et al., 2008 [42].

### Locomotor cell

Mice were habituated to the locomotor arena (27.5 cm^2^, TruScan) for 10 min and then allowed to explore freely for 30 min. Parameters measured included floor plane movement, vertical plane movement and stereotypic movement. To investigate the effect of methylphenidate (MPH) on locomotor activity, a cohort of 20 WT/20 *Brinp1*^−/−^ mice were divided into four groups: (i) WT MPH, (ii) WT saline, (iii) *Brinp1*^−/−^ MPH, (iv) Brinp1^−/−^ saline. Mice were placed in the locomotor cell for a 15 min habitation phase, then injected by acute intraperitoneal (IP) administration. Locomotor activity was recorded at 5 min intervals for 60 min post injection. Doses of 1.25 and 2.5 mg/kg MPH or saline were trialled. Testing was carried out blind of genotype and drug administration.

### Elevated plus maze

Mice were placed on an elevated platform (material: Perspex, colour: beige) at a height of 40 cm above the floor. The platform comprised of two open arms and two closed arms (each 4.5 cm wide, 30 cm in length), connected by a central square (6 cm × 6 cm). The two closed arms were protected by a 15 cm high wall. Mice were placed on the centre square and video recorded whilst exploring the maze for 5 min. Time and frequency of entry into each arm was recorded; tracking software: Noldus Ethovision 3.0.

### Y-maze

Mice were tested in two trials of a plexiglass Y-maze (material: Perspex, colour: grey) with each of the three arms having a distinctive visual cue at the end. Dimensions of each arm were 30 cm × 10 cm, with a triangle centre zone of 10 cm equal sides. Mice received a random association between visual cues and arm location. In trial 1, a partition blocked off the left arm of the maze. The mouse was placed at the end of the home arm, facing away from the centre. The time spent in each of the two available arms was recorded over 10 min. Mice were rested for 2 h. In trial 2, testing was repeated in a second trial with the partition removed and all three arms made accessible. Each of the two trials lasted 10 min; tracking software: Noldus Ethovision XT 5.0.

### Acoustic startle and pre-pulse inhibition (PPI)

Mice were placed individually inside a Perspex cylinder, closed at both ends. The cylinder was placed upon a platform sensitive to weight displacement, within a sound attenuating box with a background sound level (San Diego Instruments Startle Response System). The background white noise level was set to 70 dB. To measure acoustic startle, a strong 40 ms startle sound was played and startle response was measured by the jumping reflex (<1 s) as weight displacement on the platform. Pre-pulse inhibition was measured as the percentage reduction in startle response when a non-startling 20 ms pre-pulse of (a) 4 dB, (b) 8 dB, or (c) 16 dB above the 70 dB background sound was played 100 ms prior to the startle sound.

### Self-directed digging and grooming behaviours

Mice were placed in a plexiglass test cage (40 × 40 × 35 cm) with clean sawdust covering the base. Self-directed behaviour was video recorded for 20 min from first introduction into the novel cage. Videos were scored manually for duration and frequency of grooming and digging behaviours.

### Morris water maze

The test was performed as described by Vorhees and Williams, 2006. Briefly, a 1.9 m diameter pool was filled to a depth of 30 cm with 25 °C of water. A 15 cm diameter platform was submerged 1 cm below water level and approximately 500 ml of non-toxic white paint added to hide the platform from the animal. The test mouse was placed in a random quadrant (North, South, East or West) and allowed 2 min to find the platform. Once the platform was found, the mouse was allowed to remain there for 30 s before being removed. If the platform was not found within 2 min, the mice were guided to the platform. Mice received four training trials per day, each with a different start point, for six consecutive days; tracking software: Noldus Ethovision 3.0.

### Statistical analysis of behavioural tests

Behavioural data was analysed by the unpaired Student’s *t* test or analysis of variance (ANOVA) and represented as the mean ± standard error. Statistical analysis of reproductive phenotyping was performed by chi-square test.

### Histology

Twenty-five organs per mouse were compared for 7-week-old WT and *Brinp1*^−/−^ tissue as formalin-fixed, paraffin wax-embedded sections (10 μm), H&E staining, by the Australian Phenomics Network (http://www.australianphenomics.org.au/). Histopathological assessments of two male and two female mice, and clinical haematological analysis of one male and one female mouse, were performed. The following organs were examined for macromorphological abnormalities: testes, epididymis, seminal vesicles, prostate glands, penis, preputial gland, mammary tissue, ovaries, oviducts, uterus, cervix, vagina, clitoral gland, bladder, liver, gall bladder, stomach, duodenum, jejunum, ileum, cecum, colon, mesenteric lymph node, spleen, pancreas, kidney, adrenal glands, salivary glands and regional lymph nodes, thyroids, trachea, lungs, thymus, heart, skin, tail, eyes, harderian glands, brain, spinal cord and hind leg. For haematological analysis, blood samples from 7-week-old mice, collected into EDTA tubes, were run on the Advia 2120 haematology system: giving a red blood cell count (with indices), platelet count, and a white blood cell differential by size, granularity and peroxidase absorption. Brain sections were from the forebrain, midbrain and cerebellum. E18.5 embryos were prepared for H&E staining by the same method.

For immunostaining, WT and *Brinp1*^−/−^ mice were anaesthetised, then a transcardial perfusion was performed using phosphate-buffered saline (PBS) followed by 4 % paraformaldehyde (in PBS). Dissected brains were incubated overnight in 4 % paraformaldehyde (PFA) at 4 °C, then cryoprotected with 20 % sucrose in PBS for 72 h. Brains were frozen in OCT blocks, using an isopentane bath cooled with liquid nitrogen. Coronal sections were cut to 14 μm on a cryostat and mounted onto Superfrost Plus slides (VWR). A minimum of 4 mice per genotype were analysed, and 3–4 sections per mouse were considered for quantitative analysis. The following antibodies were used: mouse anti-NeuN (1:100, Millipore), mouse anti-parvalbumin (1:250, Sigma), rabbit anti-glial fibrillary acidic protein (anti-GFAP) (1:200, DAKO), rabbit anti-Cux1 (1:100, Santa Cruz), rabbit anti-calretinin (1:200, Swant) and rabbit anti-somatostatin (1:200, Millipore).

### Bromodeoxyuridine (BrdU) labelling

Heterozygous dams at embryonic day 12.5 (E12.5), day 14.5 (E14.5) and day 16.5 (E16.5) were injected with a single dose of BrdU solution (100 mg/kg). Pregnant dams were killed at day 18.5 of gestation, and embryos were harvested. Embryo brains were drop fixed in 4 % PFA for 12 h at 4 °C then cryopreserved in 20 % sucrose solution. WT and *Brinp1*^−/−^ embryo brains were cryopreserved in OCT medium and sectioned to 14 μm. For immunostaining of BrdU, slides were incubated in 1:7 HCL: PBS (37 % concentrated stock) at 37 °C for 1 h. Slides were then washed in PBS and permeabilized with PBS-0.1 % Triton X-100, then blocked with 10 % NGS/PBS-Triton for 30 min. A BrdU antibody (1:100, BD) was applied at RT for 2 h and counter stained with DAPI. Sections were also stained with the following antibodies: rabbit anti-Ki67 (1:1000, Leica), rabbit anti-pHH3 (1:400, Millipore) and rabbit anti-caspase 3 (1:1000, R&D systems).

Images for all immunostaining were obtained using a Nikon C1 confocal microscope. Cell count analysis were performed blind of genotype using Imaris 7.6.3. A minimum of three representative sections per mouse were analysed by dividing cortical regions into equal bins. Repeat measures two-way ANOVAs were performed for comparisons between WT and knock-out tissue. Statistical tests were performed with GraphPad Prism 5 (GraphPad).

## Results

### Generation of *Brinp1* knock-out mice

**Fig. 2:**
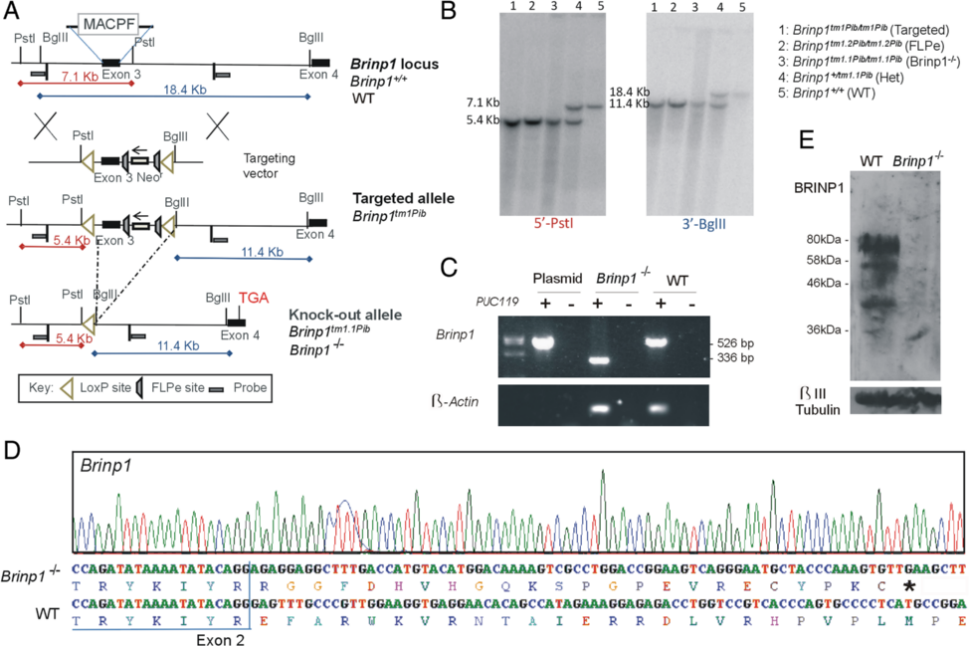
*Brinp1* targeting. **a** The *Brinp1* targeting vector was designed with a neomycin resistance cassette after exon 3, and FRT sites positioned before and after the neo^r^ cassette. The 190 bp third exon of *Brinp1* contains the start of the MACPF domain. LoxP sites flank exon 3 and the neo^r^ cassette. When crossed with a mouse line expressing Cre-recombinase, the recombination of LoxP sites resulted in the deletion of exon 3 and the neo^r^ cassette. **b** Genomic DNA isolated from the spleen was cleaved with *Pst* I and *Bgl* III and hybridised to 500 bp genomic DNA probes from the 5′ region (*Pst* I) and 3′ region (*Bgl* III) of the targeting construct. In wild-type DNA, species of 7.1 kb (*Pst* I) and 18.4 kb (*Bgl* III) were detected. These products were not present in DNA from *Brinp1* ^−/−^ mutants, replaced with shorter species of 5.4 kb (*Pst* I) and 11.4 kb (*Bgl* III). **c** cDNA from the brain tissue of WT or *Brinp1* ^−/−^ mice was tested for exon 3 deletion by RT-PCR. Primers designed to regions of exon 2 and exon 6 resulted in a PCR product size corresponding to the removal of the 190 bp exon 3 for *Brinp1* ^−/−^ cDNA. **d** Sequencing of the *Brinp1* ^−/−^ allele RT-PCR product showed the expected absence of exon 3, and that splicing fuses exons 2 and 4, resulting in a frame shift that introduces a stop codon after 24 residues. **e** By immunoblotting, full-length 85 kDa BRINP1 was present in lysates of mouse brains at postnatal day 12 and absent in *Brinp1* ^−/−^ mice

A conditional *Brinp1*-targeted allele (*Brinp1*^*tm1/Pib*^) was designed to allow Cre-recombinase-mediated, tissue-specific deletion of *Brinp1* (Fig. 2a). In the first instance, mice lacking *Brinp1* in all tissues were generated by breeding animals carrying the targeted allele with animals expressing Cre-recombinase from the two-cell embryonic stage onwards (global Cre deleter). Progeny exhibiting deletion of the selection cassette and third exon of *Brinp1* (*Brinp1*^*tm1.1/Pib*^) were inter-bred to generate homozygous *Brinp1*^*tm1.1Pib/tm1.1Pib*^ (*Brinp1*^*−/−*^) animals and WT littermates. Correct targeting and deletion of exon 3 was confirmed by Southern analysis (Fig. 2b), RT-PCR (Fig. 2c) and DNA sequencing of RT-PCR products (Fig. 2d). The resultant messenger RNA (mRNA) lacking exon 3 (*Brinp1*Δe3) reflects forced splicing between the intron 2 donor and intron 4 acceptor, fusing exons 2 and 4, and changing the reading frame to introduce a truncating stop codon (Fig. 2d). The predicted mutant protein would comprise the cleavable signal peptide (19 aa) and 54 aa of the 741 aa mature BRINP1 protein and contain no recognisable functional domains. Hence, this 54 aa form is missing over 93 % of the BRINP1 amino acid sequence and would be highly unlikely to fold correctly. It is therefore likely degraded shortly after synthesis, which is the generally accepted fate of truncated or misfolded proteins [43]. Importantly, the absence of BRINP1 protein in the *Brinp1*^*−/−*^ mouse brain was demonstrated by immunoblotting (Fig. 2e).

### *Brinp1* knock-out mice exhibit decreased postnatal survival and a reduced body weight

*Brinp1*^*−/−*^ mice born from heterozygous breeders were not observed at the expected Mendelian frequency. Only 10 out of 75 (13 %) of surviving pups were *Brinp1*^*−/−*^, indicating that half of the *Brinp1*^*−/−*^ mice died in utero or did not survive to weaning. Analysis of 18 full-term embryos showed that *Brinp1*^*−/−*^ foetuses were present at expected frequencies (6 out of 18, 33 %). We therefore conclude that *Brinp1*^−/−^ mice have impaired postnatal viability.

**Fig. 3:**
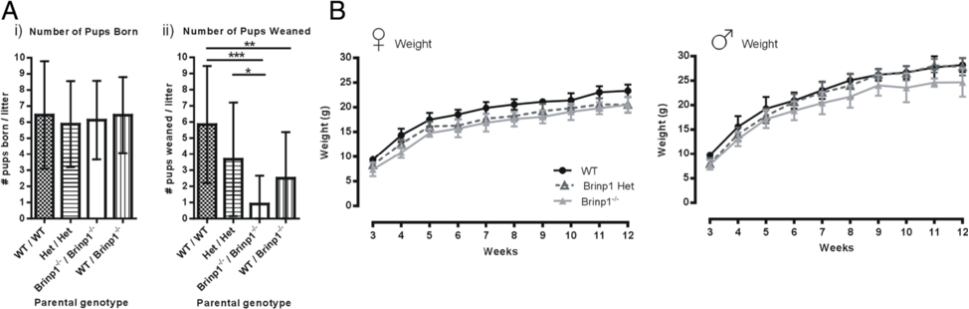
Reduced litter survival and postnatal growth of *Brinp1* knock-out mice. **a** Breeders were monitored for litter size at birth and at age of weaning (P21). *i)* No significant differences in number of pups per litter at postnatal day 0, from WT × WT, het × het, *Brinp1* ^−/−^ × *Brinp1* ^−/−^, or WT × *Brinp1* ^−/−^ parents, *F*(3,62) = 0.1624, *p* = 0.9212, one-way ANOVA. *ii)* The knock-out allele present in breeders impacted the number of pups weaned at postnatal day 21, *F*(3,62) = 9.119, *p* < 0.001, one-way ANOVA. Tukey HSD multiple comparison tests showed significant differences: WT × WT and het × het: *p* = 0.122, WT × WT and *Brinp1* ^−/−^ × *Brinp1* ^−/−^: *p* < 0.001, WT × WT and WT × *Brinp1* ^−/−^: *p* = 0.006, het × het and *Brinp1* ^−/−^ × *Brinp1* ^−/−^: *p* = 0.018, het × het and WT × *Brinp1* ^−/−^: *p* = 0.588, *Brinp1* ^−/−^ × *Brinp1* ^−/−^ and WT × *Brinp1* ^−/−^: *p* = 0.377. **b** *Brinp1* ^*−/−*^ mice weighed from week 3 to week 12. *i)* Female *Brinp1* ^−/−^ mice showed a significant reduction in body weight by repeat measures two-way ANOVA: *F*(2,18) = 27.580, *p* < 0.001. A Tukey HSD multiple comparison test found female *Brinp1* ^−/−^ mice to weight significantly less than WT littermates of the same sex (*p* < 0.001). *Brinp1* het female mice were also found to weigh significantly less than WT (*p* < 0.001). No significant effect of genotype was found between female *Brinp1* het and *Brinp1* ^−/−^ mice (*p* = 0.362). *ii)* Male *Brinp1* ^−/−^ mice also show a significant reduction in body weight by repeat measures two-way ANOVA: *F*(2,23) = 8.312, *p* = 0.002. A Tukey HSD multiple comparison test found a significant difference between male WT and *Brinp1* ^−/−^ mice (*p* = 0.002) and male *Brinp1* het and *Brinp1* ^−/−^ mice (*p* = 0.013). No significant effect of genotype found between male WT and *Brinp1* het mice (*p* = 0.704). Results represented as the mean ± SD *p < 0.05, **p < 0.0.1, ***p < 0.001, ****p < 0.0001

To investigate the reduced survival of *Brinp1*^*−/−*^ mice, the fecundity of WT × WT, het × het, *Brinp1*^*−/−*^ × *Brinp1*^*−/−*^ and WT × *Brinp1*^*−/−*^ breeding pairs was compared (Table 1). Litter numbers at birth were normal for all breeder genotypes, with no delay in first breeding or time between litters. However, post-partum survival of progeny of *Brinp1*^*−/−*^ × *Brinp1*^*−/−*^ breeders was significantly reduced (Fig. 3a) with an average of only one mouse surviving per litter after 3 days. Litter survival from het × het breeders was also reduced (average of four mice surviving per litter). Specifically, only 19/98 (22 %) of all pups born to *Brinp1*^*−/−*^ × *Brinp1*^*−/−*^ parents, and 75 % born to het × het parents, survived longer than 3 days. This was in contrast to a 91 % survival rate of progeny from WT × WT breeders.

**Table 1:** Reproductive phenotyping

		Male/Female		
	WT/WT	Het/Het	*Brinp1* ^*−/−*^/*Brinp1* ^*−/−*^	WT/*Brinp1* ^*−/−*^
Days from mating to first litter	25.8	22.8	25.0	24
Days between litters	26.7	22.1	27.6	27.4
Number of litters	19	22	20	20
Number of pups born (P0)	116	100	98	96
Number of pups weaned (P21)****	105	75	19	50
% survival	91 %	75 %	22 %	52 %

Four breeders per genotype (WT/WT, Het/Het, *Brinp1* ^*−/−*^/*Brinp1* ^*−/−*^ and WT ♂/*Brinp1* ^*−/−*^ ♀) were monitored over four months to investigate reproductive rates and postnatal survival. Whilst there were no significant differences in the numbers of pups born at postnatal day 0 (P0): *X* ^2^ (3, *n* = 410) = 2.449, *p* = 0.4846, chi-square test, the survival of mice to age of weaning (P21) was significantly impacted by the *Brinp1-*deleted allele

****X ^2^ (3, *n* = 249) = 64.43, *p* < 0.0001; chi-square test

The offspring of *Brinp1*^*−/−*^ × *Brinp1*^*−/−*^ parents were observed post-partum. Although pups survived birth and were of normal appearance, most were found dead 12–48 h later. Little to no milk was present in the stomach of the dead animals, indicating lack of adequate nutrition as a likely cause of death. Taken together, these results suggest that mothers lacking *Brinp1* are deficient in postnatal care of their offspring, resulting in neonatal death. The higher pup survival rate when a *Brinp1*^*−/−*^ female was mated with a WT male indicates that paternal care also contributes to postnatal survival.

Weekly weighing of pups from heterozygous parents revealed that *Brinp1*^*−/−*^ mice have impaired postnatal growth (Fig. 3b). These mice exhibited a significant delay in weight gain from age of first weighing (week 3) until adulthood in both male and females. Heterozygous females showed delayed weight gain close to that of *Brinp1*^*−/−*^ mice, whereas heterozygous male mice were smaller as juveniles, but their weight recovered close to WT. Despite a 10 % reduction in adult body weight, *Brinp1*^−/−^ mice had normal body length and normal brain size (data not shown).

A full pathological examination of mice at embryonic day 18.5 (E18.5) and 7-week-old (P49) *Brinp1*^−/−^ mice showed normal organ development (25 organs examined) including normal structures in the brain and spinal cord. Embryos were of a normal size and showed no developmental defects. Adult brains appeared symmetrical, with normal myelination and no ventricular dilation observed.

### *Brinp1* knock-out mice behaviour

To evaluate the effect of *Brinp1* loss on neurological function, the behaviour of *Brinp1*^−/−^ mice was assessed. In an initial screen, *Brinp1*^−/−^ mice showed normal auditory, visual and olfactory capabilities, and normal motor co-ordination on the Rotarod (Additional file 1: Figure S1a–c). No depressive-like behaviours were indicated via the tail-suspension test.

### *Brinp1* knock-out mice exhibit reduced sociability and altered ultrasonic vocalisation (USV)

**Fig. 4:**
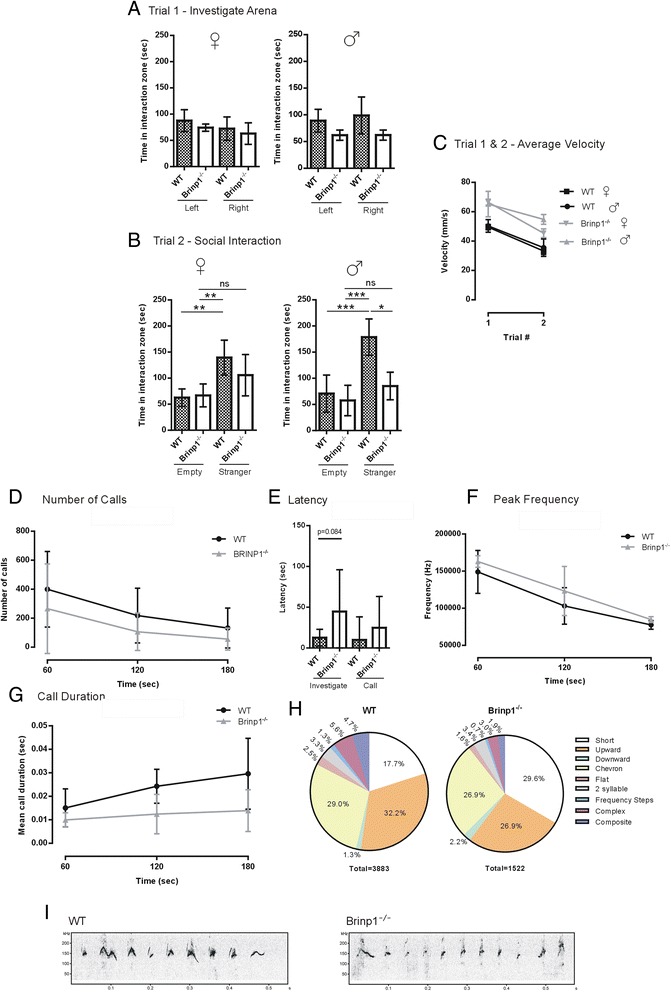
Altered social interaction and vocalisation of *Brinp1* ^−/−^ knock-out mice. **a** Habituation trial of the three-chamber social interaction test, showing interaction time between empty cages. A significant effect of genotype was observed by one-way ANOVA for male *Brinp1* ^−/−^ mice only. Female: *F*(3,19) = 1.482, *p* = 0.257, male: *F*(3,19) = 3.428, *p* = 0.029. **b** *Brinp1* ^−/−^ mice show reduced interaction time with a sex-matched novel mouse. A significant effect of genotype was observed by one-way ANOVA; female: *F*(3,19) = 7.542, *p* = 0.002, male: *F*(3,19) = 15.07, *p* < 0.0001. A Tukey HSD post hoc test showed significant differences between the intruder mouse and empty cage for WT but not *Brinp1* ^−/−^ mice. Female: WT empty – WT stranger: *p* = 0.004, *Brinp1* ^*−/−*^ empty – *Brinp1* ^*−/−*^ stranger: *p* = 0.199, WT empty – *Brinp1* ^*−/−*^ empty: *p* = 0.996, WT stranger – *Brinp1* ^*−/−*^ stranger: *p* = 0.304, WT empty – *Brinp1* ^*−/−*^ stranger: *p* = 0.136, WT stranger – *Brinp1* ^*−/−*^ empty: *p* = 0.006. Male: WT empty – WT stranger: *p* < 0.001, *Brinp1* ^*−/−*^ empty – *Brinp1* ^*−/−*^ stranger: *p* = 0.528, WT empty – *Brinp1* ^*−/−*^ empty: *p* = 0.910, WT stranger – *Brinp1* ^*−/−*^ stranger: *p* = 0.01, WT empty – *Brinp1* ^*−/−*^ stranger: *p* = 0.887, WT stranger – *Brinp1* ^*−/−*^ empty: *p* < 0.001. **c** *Brinp1* ^−/−^ mice exhibited hyperactivity in both trials of three-chamber social interaction test; female: *F*(1,8) = 55.69, *p* < 0.001, male: *F*(1,8) = 41.08, *p* < 0.001, repeat measures two-way ANOVA. **d** Ultrasonic vocalisation (USV) of adult male mice: Number of calls, divided into 1 min bins, *F*(1,17) = 1.693, *p* = 0.21, repeat measures two-way ANOVA. **e** Latency of male *Brinp1* ^−/−^ mice to investigate cotton bud (nose <1 cm from bud), *t*(17) = 1.835, *p* = 0.084, and latency to call *t*(17) = 0.9709, *p* = 0.3452, unpaired Student’s *t* tests. **f** Peak call frequency of USVs from male *Brinp1* ^−/−^ mice, divided into 1 min bins. No significant effect of genotype was observed: *F*(1,17) = 3.39, *p* = 0.089, repeat measures two-way ANOVA. **g** Male *Brinp1* ^−/−^ mice emit shorter USV calls. Call duration data was divided into 1 min bins. *F*(1,17) = 8.17, *p* = 0.014, repeat measures ANOVA. **h** Pie chart showing distribution of call types of male *Brinp1* ^−/−^ mice. For designation of call-type categories, refer to Scattoni et al. [42]. Unidentified calls (11.7 % WT, 11.3 % *Brinp1* ^−/−^) were excluded. **i** Representative spectrogram of first calls from a WT and a male *Brinp1* ^−/−^ mouse. *p < 0.05, **p < 0.01, ***p < 0.001 ****p < 0.0001. N = 6 females, 6 males per genotype for sociability experiment (A-C). N = 10 WT, 9 *Brinp1-/- *male mice for USV experiment (D-I). All data represented as the mean ± SD

A pronounced decrease in sociability was observed by the *Brinp1*^−/−^ mice tested for social interaction with an unfamiliar mouse. In the first trial of the three-chamber social interaction test, *Brinp1*^−/−^ and WT mice showed no preference between the left and right empty cages. Male *Brinp1*^−/−^ mice spent slightly less time investigating the empty cages, which may indicate altered exploratory behaviour (Fig. 4a). In the second trial, when presented with a stranger mouse in one of the cages, *Brinp1*^−/−^ mice spent significantly less time interacting with the intruder, whilst showing no difference in interaction time with the empty cage (Fig. 4b). Brinp1^−/−^ mice also exhibited hyperactivity during both trials (Fig. 4c). Results for this test were reproduced with a second cohort (*n* = 12 mice per genotype, *p* < 0.05) which also showed reduced time interacting with a stranger mouse.

Communication by *Brinp1*^−/−^ mice was investigated by analysing the ultrasonic vocalisation (USV) calls of male mice presented with pheromones from estrus or proestrous female mice [44]. Compared to WT, *Brinp1*^−/−^ mice showed a trend towards reduced number of calls, a longer latency to call and longer latency to investigate the cotton bud, but these parameters did not reach the 95 % confidence level (Fig. 4d, e). No change in call amplitude was evident, although a trend of increased peak frequency of calls was observed (*p* = 0.089) (Fig. 4f). Notably, *Brinp1*^−/−^ mice showed significantly shorter call durations (*p* < 0.05) (Fig. 4g). This reduced call length reflected changes to the distribution of call types. *Brinp1*^−/−^ mice emitted a higher percentage of ‘short’ calls and lower percentage of ‘complex’ or ‘composite’ longer calls (Fig. 4h, i). This reduction in complex and composite calls indicates that *Brinp1* influences vocalisation.

### *Brinp1* knock-out mice are hyperactive and exhibit changes in exploratory behaviour

**Fig. 5:**
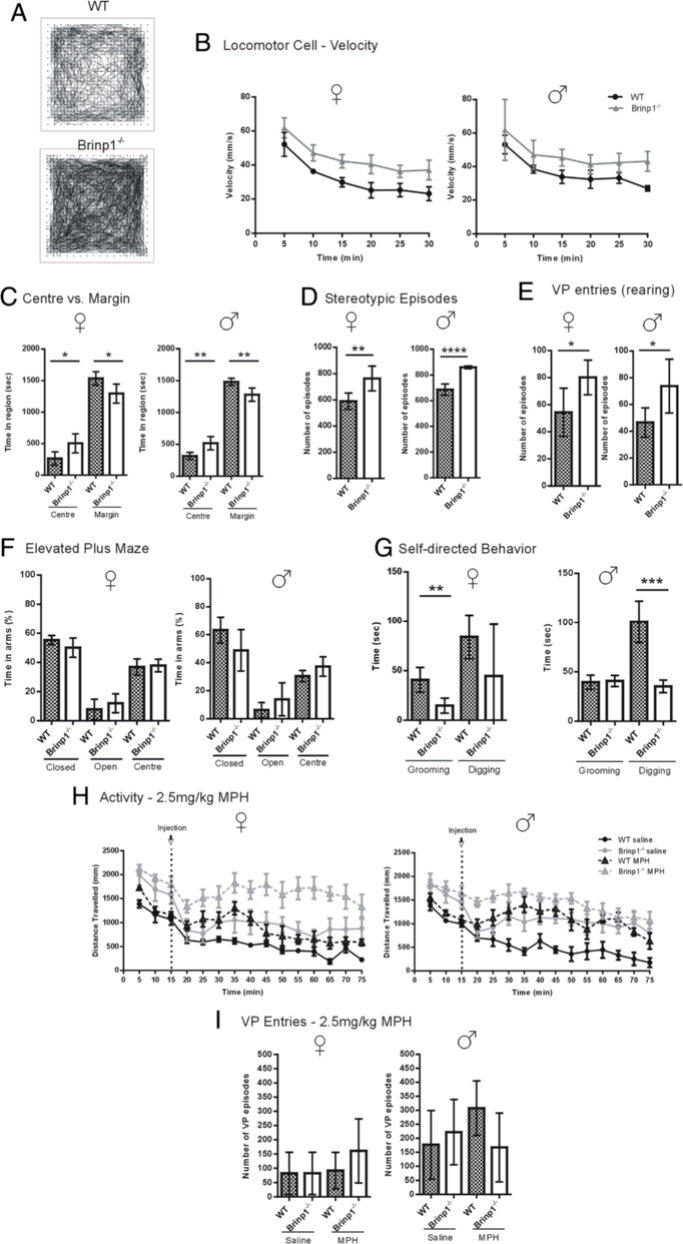
*Brinp1* knock-out mice exhibit hyperactivity and altered exploratory behaviour. **a** Increased locomotor activity of *Brinp1* ^−/−^ mice in a 30 min trial in a locomotor cell, shown as a representative data trace for male WT and *Brinp1* ^−/−^ litter mates. **b** Increased velocity of *Brinp1* ^−/−^ mice in the locomotor cell; female: *F*(1,8) = 27.00, *p* < 0.001, male: *F*(1,8) = 8.25, *p* = 0.021, repeat measures two-way ANOVA. **c** Increased exploratory behaviour of *Brinp1* ^−/−^ mice in a locomotor cell, shown as increased time in the centre and reduced time in marginal areas; female: *t*(8) = 2.919, *p* = 0.0193, male: *t*(8)=5.043, *p* = 0.0054, unpaired Student’s *t* tests. **d** *Brinp1* ^−/−^ mice showed increased stereotypic episodes in the locomotor cells; female: *t*(8) = 3.414, *p* = 0.0092, male: *t*(8) = 3.772, *p* < 0.0001, unpaired Student’s *t* tests. **e** *Brinp1* ^−/−^ mice exhibited increased rearing behaviour, measured as number of vertical plane (VP) entries; female: *t*(8) = 2.630, *p* = 0.0302, male: *t*(8) = 2.662, *p* = 0.0287. **f** Elevated plus maze: male *Brinp1* ^−/−^ mice spent more time in the open arms relative to the closed arms of the maze, indicating reduced anxiety. Analysis by repeat measures ANOVA shows a genotype × arm interaction for male mice: *F*(2,20) = 3.525, *p* = 0.49. Female genotype × arm interaction: *F*(2,20) = 1.417, *p* = 0.266. **g** *Brinp1* ^−/−^ mice self-directed behaviour: *Brinp1* ^−/−^ mice were observed for self-directed behaviour in a test plexiglass cage lined with sawdust for 20 min. Male *Brinp1* ^−/−^ mice showed a decrease in time digging: *t*(8) = 5.765, *p* = 0.0004, whilst female *Brinp1* ^−/−^ mice exhibited decreased grooming duration *t*(8) = 3.977, *p* = 0.0041, unpaired Student’s *t* tests. **h** WT and *Brinp1* ^−/−^ mice locomotor activity following injection of MPH/saline in the locomotor cell. An acute IP injection of 2.5 mg/kg of MPH increased locomotor activity for both WT and *Brinp1* ^−/−^ mice, *N* = 10 WT MPH, 10 *Brinp1* ^−/−^ MPH, 10 WT saline, 10 *Brinp1* ^−/−^ saline. Repeat measures two-way ANOVA analysis, performed on number of distance travelled post injection (20 min+) revealed an effect of genotype; female: *F*(1,16) = 22.427, *p* < 0.001, male: *F*(1,16) = 19.922, *p* < 0.001, and an effect of 2.5 mg/kg drug treatment; female: *F*(1,16) = 13.962, *p* = 0.002, male *F*(1,16) = 19.239, *p* < 0.001, whereby administration of MPH resulted in a significant increase in distance travelled for both WT and *Brinp1* ^*−/−*^ mice of both sexes. **i** A dose of 2.5 mg/kg MPH did not significantly alter the number of rearing episodes of *Brinp1* ^−/−^ mice post drug administration; female: *F*(3,16) = 0.5698, *p* = 0.3961, male: *F*(3, 16) = 0.1351, *p* = 0.2403, one-way ANOVA. *N* = 5 female, 5 male mice per genotype for locomotor cell (A-E) and digging/grooming (G) experiments. *N* = 6 female, 6 male mice per genotype for EPM experiment (F). *P<0.05, **P<0.01, ***P<0.001,****P<0.0001

Hyperactivity of *Brinp1*^−/−^ mice was revealed by testing animals in the locomotor cell where, over a period of 30 min, the *Brinp1*^−/−^ mice travelled 50 % further, spent less time resting and showed a consistent increase in velocity compared to WT (Fig. 5a, b). *Brinp1*^−/−^ mice also spent proportionally more time in the centre of the locomotor cell (Fig. 5c). Notably, *Brinp1*^−/−^ mice showed an increase in stereotypic episodes (repeat motion within a small area) and increased rearing (vertical plane activity) (Fig. 5d, e).

**Fig. 6:**
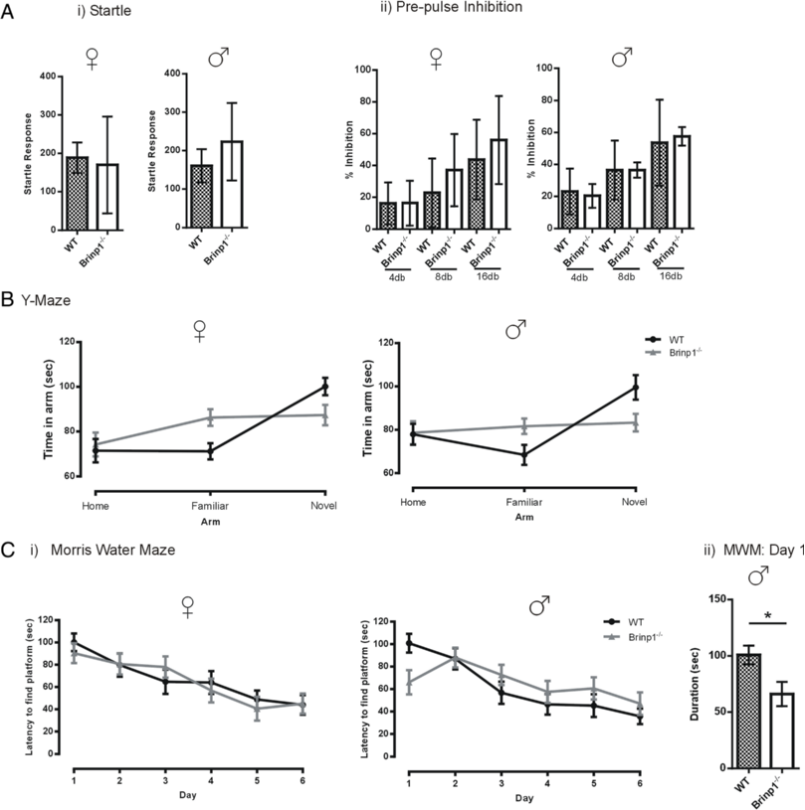
*Brinp1* knock-out mice show normal PPI and impaired short-term memory. **a**. *Brinp1* ^−/−^ mice showed *i)* normal startle response; female: *t*(10) = 0.288, *p* = 0.779, male: *t*(10) = 1.045, *p* = 0.321, unpaired Student’s *t* test, and *ii)* and no significant effect of genotype on pre-pulse inhibition (PPI), female: *F*(1,10) = 0.695, *p* = 0.424, male: *F*(1,10) = 0.003, *p* = 0.958, repeat measures two-way ANOVA. *N* = 6 female, 6 male mice per genotype. **b** Y-maze: Results from two cohorts tested indicate that Brinp1−/− mice did not display an increase in time spent exploring the novel arm, in comparison to WT controls. Analysis by repeat measures two-way ANOVA revealed a significant interaction effect for genotype × arm; female: *F*(2,40) = 3.829, *p* = 0.030, male: *F*(2,40) = 3.737, *p* = 0.033. *N* = 11 female, 11 male mice per genotype. Data presented as the mean ± SE. **c** *Brinp1* ^−/−^ mice showed no impairment in learning the location of a hidden platform in the Morris water maze: *i)* no significant main effect of genotype on time locating the platform; female: *F*(1,9) = 0.530, *p* = 0.819, male: *F*(1,9) = 0.160, *p* = 692. *N* = 5 female, 5 male mice per genotype. Data presented as the mean ± SE. *ii)* Male *Brinp1* ^−/−^ mice showed an improvement in locating the platform on day 1, as a decrease in time to find the platform, *t*(38) = 2.549, *p* = 0.0173, unpaired Student’s *t* tests. Data presented as the mean ± SD except where otherwise stated. *p < 0.05, **p < 0.0.1, ***p < 0.001, ****p < 0.0001

To examine whether the absence of *Brinp1* affects anxiety-like behaviour, mice were allowed to freely explore an elevated plus maze for 5 min. Testing of the *Brinp1*^−/−^ mice on the maze showed male *Brinp1*^−/−^ mice spent significantly less time in a closed arm. (Fig. f). The number of entries into each arm of the EPM was unaffected (Additional file 1: Figure S1d).

*Brinp1*^−/−^ mice showed a normal preference for investigating an unfamiliar object in a novel object recognition test (Additional file 1: Figure S1f). To assess mice in an environment similar to their home cage, mice were placed in a sawdust-lined, plexiglass box. Mice in this environment showed increased rearing behaviour at a similar frequency to the locomotor cell. In addition, there was a decrease in the amount of time the *Brinp1*^−/−^ mice spent digging compared to wild type (Fig. 5g). Male mice showed no significant differences in grooming behaviour, whilst female mice showed significantly decreased grooming (Fig. 5g).

### Methylphenidate does not decrease hyperactivity of *Brinp1* knock-out mice 

Methylphenidate (MPH) is a psychostimulant drug commonly used to treat hyperactivity and inattention in children diagnosed with ADHD [45]. It has previously been shown to reduce hyperactivity in mouse models of ADHD [46, 47]. To investigate whether MPH reduces hyperactivity of *Brinp1*^−/−^ mice, animals were tested using a published regime that previously measured activity in the locomotor cell of spontaneous SNAP25 mutant Colombomo mice [48]. Activity was determined as the distance travelled, measured at 5 min intervals. In the habituation phase prior to injection, *Brinp1*^−/−^ mice travelled significantly further than WT mice (Fig. 5h). The effect of MPH (2.5 mg/kg) as a stimulant was indicated by the expected increase in activity of the WT animals , peaking at 20 min post injection. This pattern of activity was comparable to that of the saline-treated *Brinp1*^−/−^ group. The *Brinp1*^−/−^ MPH group also showed an increase in locomotor activity above the level of both the *Brinp1*^−/−^ saline and WT MPH groups. Results at 1.25 mg/kg MPH showed a milder effect of increased activity for both the WT and *Brinp1*^−/−^ groups (Additional file 1: Figure S1f). Methylphenidate did not significantly alter rearing activity at doses of 2.5 mg/kg (Fig. 5i) or 1.25 mg/kg. Overall these results demonstrate that at two doses, MPH stimulates activity in both controls and *Brinp1*^−/−^ animals, and does not ameliorate hyperactivity of the knock-out animals. This is a similar response to the drug tested with SNAP25 mutant mice [48].

### *Brinp1* knock-out mice exhibit normal sensory gating

The pre-pulse inhibition (PPI) test is used to measure sensory gating in mice, which models deficits in human subjects diagnosed with schizophrenia. No abnormalities in startle response or sensory gating for *Brinp1*^−/−^ mice were detected by startle/pre-pulse inhibition testing, indicating that these mice do not model this aspect of human schizophrenia (Fig. 6a).

### *Brinp1* knock-out mice have impaired short-term memory

*Brinp1*^−/−^ mice were tested for spatial learning and memory in the Y-maze test (Fig. 6b). With a 2 h interval between trials, *Brinp1*^−/−^ mice did not show the typical increase in time spent exploring a novel arm, indicating impaired short-term memory. To determine whether long-term memory impairment was also evident, *Brinp1*^−/−^ mice were tested using the Morris water maze. *Brinp1*^*−/−*^ mice showed no impairment in this test (Fig. 6c). Possibly due to their hyperactivity, male knock-out mice showed faster time, as well as a shorter swim distances (*p* = 0.048), required to find the platform compared to WT on day 1 of the experiment.

### *Brinp1* knock-out mice show an increase in PV-positive interneurons in the somatosensory cortex

**Fig. 7:**
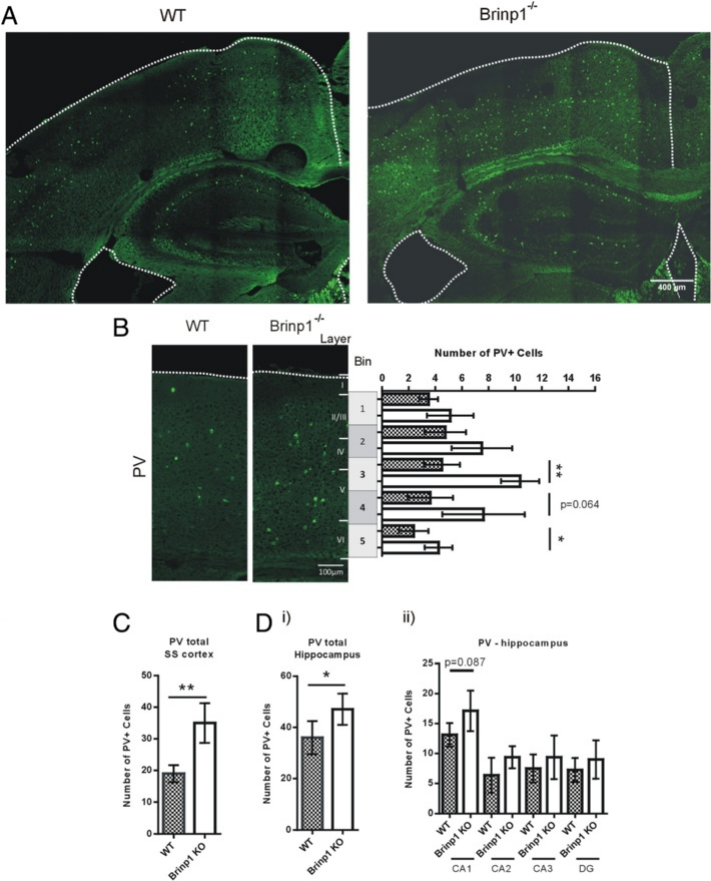
Increased PV+ interneuron cell density in the adult *Brinp1* knock-out neocortex and hippocampus. **a** Representative coronal section showing parvalbumin (PV)-positive interneurons in the neocortex and medial hippocampus of WT and *Brinp1* ^−/−^ mice. **b** An increase in number of PV-positive cells in bins 3 and 5, corresponding with layers IV–VI of the somatosensory neocortex of *Brinp1* ^−/−^ mice, *F*(6) = 21.602, *p* = 0.004, repeat measures two-way ANOVA. **c** An increase in total numbers of PV-positive cells was observed in the somatosensory neocortex of *Brinp1* ^−/−^ mice, *t*(6) = 4.684, *p* = 0.0034. **d** PV-positive interneurons in the hippocampus: *i)* an increase in total numbers of PV-positive cells in the hippocampus of *Brinp1* ^−/−^ mice *t*(6) = 2.497, *p* = 0.0467. *ii)* A near significant difference was observed for the CA1 region, *p* = 0.087, whilst no significant differences were observed for CA2, CA3 or dentate gyrus regions. *N *= 4 mice per genotype

Brains from adult WT and *Brinp1*^−/−^ mice were analysed for changes in cortical anatomy and hippocampal structure, at Bregma co-ordinate −1.94, encompassing the medial hippocampus and the somatosensory cortex. Analysis with an antibody to PV, a marker of a subpopulation of interneurons, showed an increase in cellularity in layers IV and VI of the neocortex (Fig. 7a–c) in *Brinp1*^−/−^ mice, as well as an increase in total PV-positive cells in the hippocampus (Fig. 7d). Increased PV interneuron numbers were also suggested in the CA1 region of the hippocampus (*p* = 0.087). No significant changes in number or distribution of other interneuron subpopulations, marked by calretinin or somatostatin, were detected (Additional file 2: Figure S2a, b). No significant changes in pyramidal neuron layering were detected with a pan-neuronal marker (NeuN) or layer II–IV marker Cux1 (Additional file 3: Figure S3). The medial hippocampus also showed normal morphology with this antibody. Brains were stained with the global astrocyte marker, GFAP. No changes in cortex or hippocampal astrocyte numbers were observed (Additional file 2: Figure S2c).

### *Brinp1* knock-out embryos show normal cortical cell survival and proliferation

**Fig. 8:**
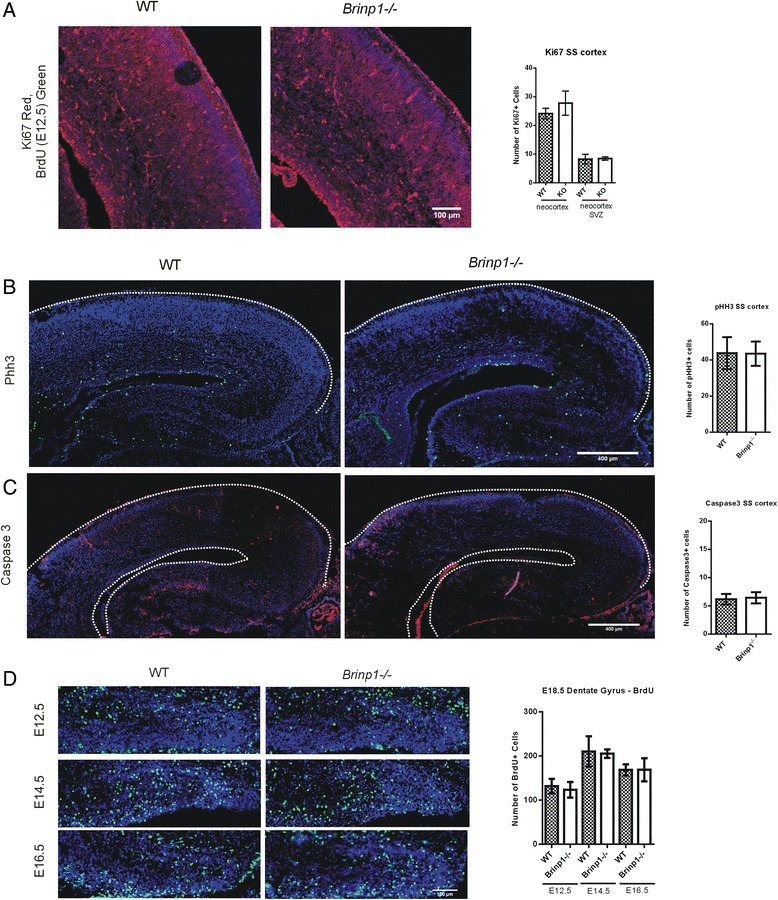
Normal cell proliferation in the E18.5 *Brinp1* knock-out mouse brain. **a** No significant change in number of Ki67+ cells (proliferation marker) in the embryonic day 18.5 (E18.5) somatosensory neocortex (coronal) of *Brinp1* ^*−/−*^ mice. Neocortex: *t*(8) = 1.768, *p* = 0.1150, neocortex SVZ: *t*(8) = 0.2736, *p* = 0.7913, unpaired Student’s *t* tests. *N* = 5 WT and 5 *Brinp1* ^*−/−*^ mice. **b** No significant change in number of Phh3+ cells (a marker of cells undergoing mitosis) in the neocortex or hippocampus of E18.5 *Brinp1* ^*−/−*^ brains (coronal sections). Neocortex SVZ: *t*(6) = 0.1509, *p* = 0.8850, neocortex: *t*(6) = 0.04147, *p* = 0.9683, hippocampus SVZ: *t*(6) = 0.1211, *p* = 0.9083, hippocampus: *t*(6) = 2.008, *p* = 0.0914, unpaired Student’s *t* tests. *N* = 4 WT and 4 *Brinp1* ^*−/−*^ mice. **c** No significant change in number of activated caspase 3-positive cells (a marker of cells undergoing apoptosis) in the neocortex or hippocampus of E18.5 *Brinp1* ^*−/−*^ brain (coronal). Neocortex: *t*(7) = 0.2564, *p* = 0.8050, hippocampus: *t*(7) = 1.114, *p* = 0.3020, unpaired Student’s *t* tests. *N* = 5 WT and 4 *Brinp1* ^*−/−*^mice. **d** BrdU+ cell distribution in the E18.5 dentate gyrus following administration of BrdU to *Brinp1* heterozygous dams at the following time points: embryonic day 12.5 (E12.5), day 14.5 (E14.5) or day 16.5 (E16.5). No significant change in cell number of E12.5-, E14.5- or E16.5-born cells (BrdU-labelled) in the E18.5 dentate gyrus. E12.5 BrdU+ cell count: *t*(8) = 0.7758, *p* = 0.4602, E14.5 BrdU+ cell count: *t*(8) = 0.2668, *p* = 0.7973, E16.5 BrdU+ cell count: *t*(8) = 0.01437, *p* = 0.9890, unpaired Student’s *t* test. *N* = 5 WT and 5 *Brinp1* ^*−/−*^ mice. All images counter stained with 4′,6-diamidino-2-phenylindole (DAPI, *blue*)

*Brinp1*^−/−^ embryonic brains were examined at E18.5. No significant differences in cortical Ki67-positive cell numbers were detected, indicating that *Brinp1* does not affect cortical cell proliferation in the E18.5 mouse brain (Fig. 8a). No significant changes in the mitotic cell numbers were apparent in the cortex using a phosphohistone-H3 (Phh3) antibody, a marker of mitotic activity (Fig. 8b). There were also no significant changes in apoptosic cell numbers in the E18.5 cortex, using a caspase 3 antibody (Fig. 8c).

Layer formation during embryonic development was examined at E12.5 (layer V and VI formations), E14.5 (layer IV formation) and E16.5 (layers II/III formation). Pregnant females, from heterozygous matings, were injected with BrdU at one of each of these time points. Due to the poor postnatal survival rates of *Brinp1*^−/−^ mice, embryos were harvested just prior to birth, at E18.5. There were no significant differences in BrdU+ cell numbers detected in the subventricular zone in any of the three injection time points. There were also no significant differences in E12.5-, E14.5- or E16.5-born BrdU+ cell numbers in the dentate gyrus of the E18.5 hippocampus (Fig. 8d). Together, these results indicate that *Brinp1* has no detectable effect on the proliferation or cell survival within the neocortex or hippocampus.

### Increased expression of Astrotactin 1 and Astrotactin 2 in the developing brain of *Brinp1* knock-out mice

**Fig. 9:**
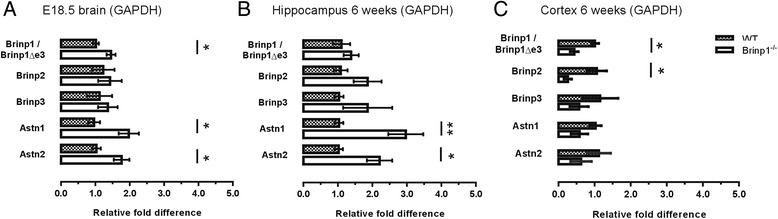
Up-regulation of Astrotactin 1 and Astrotactin 2 mRNA in the embryonic brain and adult hippocampus of *Brinp1* knock-out mice. **a** qPCR showing a significant increase in *Astn1* mRNA, *t*(9) = 2.800, *p* = 0.0207, and *Astn2* mRNA, *t*(9) = 2.829, *p* = 0.0222, unpaired Student’s *t* test, in the developing (E18.5) mouse brain. Levels of *Brinp1* exon 3-deleted (*Brinp1*Δe3) mRNA also increase, compared to levels of *Brinp1* in the WT mice: *t*(9) = 2.733, *p* = 0.0231. No significant changes overserved for *Brinp2* mRNA levels *t*(9) = 0.4109, *p* = 0.6908, or *Brinp3* mRNA levels: *t*(9) = 0.5277, *p* = 0.6105, unpaired Student’s *t* test. **b** An increase in *Astn1* and *Astn2* expression was also detectable in the hippocampus at 6 weeks of the *Brinp1* ^−/−^ mice: *Astn1:* t (9) = 3.384, *p* = 0.0081, *Astn2: t*(9) = 2.821, *p* = 0.0200, unpaired Student’s *t* tests. No significant changes were detected in levels of exon 3-deleted (*Brinp1*Δe3) mRNA *t*(9) = 0.8505, *p* = 0.4171, *Brinp2* mRNA *t*(9) = 1.616, *p* = 0.1405, or *Brinp3* mRNA *t*(9) = 1.047, *p* = 0.3222, unpaired Student’s *t* tests. **c** In the 6-week-old *Brinp1* ^−/−^ cortex, less *Brinp1* exon 3-deleted (*Brinp1*Δe3) mRNA (*t*(5) = 3.611, *p* = 0.0154) and *Brinp2* mRNA (*t*(5) = 3.113, *p* = 0.0265) were detected, with no significant change in expression of either *Astn1*, *Astn2* or *Brinp3 Astn1*: *t*(5) = 1.572, *p* = 0.1909, *Astn2*: *t*(5) = 1.139, *p* = 0.3182, *Brinp3: t*(5) = 1.133, *p* = 0.3087, unpaired Student’s *t* tests. *N* = 3 WT, 4 *Brinp1* ^*−/−*^, *p < 0.05, **p < 0.01. Normalisation against GAPDH expression levels. All data represented as the mean ± SE

Quantitative PCR was performed on mRNA from the brains of *Brinp1*^−/−^ mice, to assess whether homologous MACPF family members are up-regulated. The late embryonic brain (E18.5) showed a 2-fold increase in both *Astn1* and *Astn2* mRNA (Fig. 9a). Levels of the *Brinp1* exon 3-deleted, frameshifted mRNA (*Brinp1*Δe3) also increased by 1.5-fold compared to levels of *Brinp1* mRNA in the WT mice. No significant changes to the levels of *Brinp2* or *Brinp3* mRNA were evident at this developmental time point. At 6 weeks of age, *Brinp1*^*−/−*^ mice showed a 3-fold increase in *Astn1* mRNA and a 2-fold increase in *Astn2* mRNA in the hippocampus (Fig. 9b). Down-regulation of both *Brinp1*Δe3 and *Brinp2* mRNA was detected in the adult cortex, with no change in expression of either Astrotactin mRNA (Fig. 9c). Normalisation with *GAPDH* and *β-actin* gave similar results (*β-actin* data shown in Additional file 4: Figure S4).

## Discussion

### *Brinp1* knock-out mice model core social communication symptoms of autism spectrum disorder

Our study of *Brinp1*^−/−^ mice has revealed behavioural phenotypes that resemble social and communication impairments found within human ASD. The three-chamber social interaction test is an accepted behavioural test to investigate social deficits relevant to autism. Using this test, *Brinp1*^−/−^ mice spent significantly less time interacting with a stranger sex-matched mouse: a sign of social withdrawal. Furthermore, testing of ultrasonic vocalisations of adult male mice is a key method for behavioural phenotyping of NDDs [44, 49]. Male *Brinp1*^−/−^ mice exhibited an altered vocalisation phenotype when presented with pheromones from estrus females. The shorter call duration of *Brinp1*^−/−^ mice, with fewer complex calls, indicates altered communication ability.

*Brinp1*^−/−^ mice showed poor postnatal survival. The marked reduction in sociability and communication observed in *Brinp1*^−/−^ adults may manifest in reduced attentiveness towards litters, thereby hindering neonate survival if mothers fail to provide adequate thermal insulation, nourishment and post-parturition care towards their pups. A series of cross-fostering experiments would be required to demonstrate this. Reduced survival may also be a result of flawed two-way communication between dam and pup. Future investigation of the social communication deficits of *Brinp1*^*−/−*^ mice should determine whether *Brinp1*^*−/−*^ pups emit altered vocalisations. It would also be interesting to investigate ultrasonic vocalisation between two interacting *Brinp1*^*−/−*^ mice.

Repetitive movements such as increased grooming are reported in some but not all mouse models of ASD [50, 51]. *Brinp1*^−/−^ mice did not exhibit increased grooming. However, increased rearing and increased stereotypic episodes were observed in the locomotor cell. Whilst interesting observations, these behaviours do not necessarily model the repetitive movements of autism as such movements could also the result of hyperactivity.

Taken together, the above observations suggest that *Brinp1*^−/−^ mice model social communication aspects of human ASD, since poor sociability, delayed language and impaired communication skills are fundamental facets of the pathology.

### *Brinp1* knock-out mice are hyperactive, with short-term memory impairment

*Brinp1*^−/−^ mice are hyperactive, evidenced by a 50 % increase in horizontal plane activity in the locomotor cell. There are also some indications of changes in exploratory behaviour of *Brinp1*^−/−^ mice; showing increased rearing, and altered time spent in exposed regions of the locomotor cell, as well as less time in the protected arms of the elevated plus maze. The hyperactivity of *Brinp1*^−/−^ mice was present in multiple testing arenas, and it therefore should be noted that this may confound other aspects of behaviour, such as the mouse’s response to social approach tasks or attention during memory-based tasks.

*Brinp1*^−/−^ mice have a 10 % reduction in body mass. This may be because of increased energy expenditure due to hyperactivity, and is consistent with variants in the *Brinp1* locus at 9q33.1 in patients with growth delay [52].

Methylphenidate (Ritalin) is a psychostimulant commonly used in the treatment of ADHD symptoms [45, 53], although a considerable proportion of patients do not respond [54]. We examined the predictive validity of *Brinp1*^−/−^ mice as a model for ADHD by investigating their response to methylphenidate. Methylphenidate did not reduce the hyperactivity of *Brinp1*^−/−^ mice, indicating that *Brinp1* deletion is unlikely to directly affect the dopaminergic pathways of the prefrontal cortex, as hypothesised for ADHD aetiology [55]. A future direction would be to examine the effect of other drugs, such as *d*-amphetamine (a psychostimulant that promotes catecholamine release at pre-synaptic terminals) [48], atomoxetine (a norepinephrine reuptake inhibitor) [56] or clonidine (and α2-adrenergic receptor agonist) [57], on hyperactivity as well as sociability of *Brinp1*^−/−^ mice.

*Brinp1*^−/−^ mice did not show preference in exploring the novel arm of the Y-maze, when testing with a 2 h interval between trials, indicating impaired short-term memory. The *Brinp1*^−/−^ mice performed normally in the Morris water maze, demonstrating that long-term memory is unaffected. The short-term memory impairment of *Brinp1*^−/−^ mice may model cognitive impairment evident in a subset of autistic patients, or possibly psychiatric disorders that show association with the *BRINP1* locus [30, 33].

### *Brinp1* knock-out mice exhibit changes in PV interneurons in the neocortex

More parvalbumin-positive interneurons were detected in the adult *Brinp1*^−/−^ mouse neocortex and hippocampus. PV interneurons are a subset of GABA-ergic neurons that play an inhibitory role in the cortex. Importantly, changes in PV interneuron populations have been reported in other ASD mouse models, as well as ASD patients [14, 15]. The cell-specific increase of this subtype of interneurons is of particular interest as this implies an unknown effect of *Brinp1* deletion on fast-spiking basket and chandelier cells. We postulate that the absence of *Brinp1* causes dysregulated proliferation or migration of these specific PV interneuron populations, resulting in a greater density of such cells in defined regions of the neocortex. Further investigation is needed to determine the underlying molecular mechanism, whether the increase in PV interneurons in the somatosensory cortex is matched with increases in other cortical regions, and to determine the age at which this phenotype first appears. As cortical interneurons have a critical role in regulating network excitability generated by pyramidal interneurons [58], the gain of PV interneurons may therefore shift the balance between cortical excitation and inhibition, resulting in the reported behavioural changes. An imbalance between excitation and inhibition has been observed in the brains of humans with ASD [59–61].

Interestingly, mRNA of the MACPF family members Astrotactins 1 and 2 increased in the embryonic brain (E18.5) and 6-week-old hippocampus of *Brinp1*^−/−^ mice. Both Astrotactins 1 and 2 are reported to facilitate neuronal migration [28, 29]. This suggests that Astrotactins and *Brinp1* may have similar functions, and perhaps work in a common pathway, such that Astrotactins 1 and 2 functionally compensate for the absence of *Brinp1* during neuronal migration. The increase of *Brinp1*∆e3 mRNA in *Brinp*^*−/−*^ animals is possibly due to compensatory pressure in the absence of functional protein.

During the final stages of our study, Kobayashi et al. reported a different line of *Brinp1*^−/−^ mice, possessing a deletion in exon 8 (*Brinp1*∆e8) [22]. Our observations are consistent with some but not all of their findings. Both *Brinp1* knock-out lines exhibit increased activity, reduced sociability, reduced anxiety and impaired working memory. Importantly, however, Kobayashi et al. did not investigate communication (or report potentially associated breeding difficulties), whereas our findings reveal a profound communication deficit suggesting an ASD-like endophenotype which is consistent with reports of autism and language delay in patients with de novo human mutations at the 9q33.1 locus. Although they suggest a schizophrenia-like phenotype in the *Brinp1*Δe8 mice, Kobayashi et al. did not test pre-pulse inhibition (PPI). By contrast, we have demonstrated that PPI, used to measure sensory gating, is normal for *Brinp1*^*−/−*^ mice.

Overall, similar behavioural phenotypes were evident in both female and male *Brinp1*^−/−^ animals. However, we note some subtle gender-specific effects in *Brinp1*^−/−^ mice. For example, reduced anxiety is evident on the EPM for males only, whereas only female *Brinp1*^−/−^ mice exhibited reduced grooming behaviour. There is greater weight variability in male mice during postnatal development, whilst heterozygous mice are affected in adult females only.

Kobayashi et al. report that adult *Brinp1*∆e8 knock-out animals exhibit increased hippocampal neurogenesis and more PV interneurons in the hippocampus, concluding that the altered behaviours reflect a hippocampal-specific defect. No cortical or other perturbations were reported in the *Brinp1*∆e8 knock-out mice. By contrast, our *Brinp1* knock-out animals display no evidence of increased cell proliferation in the embryonic cortex and no evidence for increased embryonic cell proliferation. Like Kobayashi et al., we observed an increase in PV interneurons in the adult hippocampus, but this is not hippocampus-specific, as we also detected additional PV interneurons in the neocortex. Given these observations, we therefore disagree with the Kobayashi et al. conclusion that *Brinp1* is a negative regulator of general cell proliferation and that the behavioural alterations in mice lacking BRINP1 arise solely due to disruption of the hippocampus. Our findings demonstrate that changes in density of PV interneurons in the *Brinp1*^*−/−*^ neocortex and hippocampus from an unknown mechanism likely underpin the reported behavioural phenotypes.

An explanation for the differences between the two studies may lie in the differences between the two *Brinp1* mutant alleles. The Kobayashi study involved a conventionally targeted *Brinp1*^*−/−*^ line carrying a deletion of exon 8, which removes the terminal 418 residues of the 760 residue protein but retained the promoter and the potential to express a smaller protein containing the MACPF domain (No protein analysis was carried out on the *Brinp1*Δe8 mice). By contrast, our knock-out allele removes exon 3 and truncates the protein after the first 74 amino acids (preceding the MACPF domain), and immunoblotting established that BRINP1 production was abolished. Our targeting strategy also included removal of the neomycin selection cassette, which is known to have effects on survival and physiology if retained in the targeted locus [62, 63]. The *Brinp1*Δe8 allele does not have the selection cassette removed, which may influence the phenotype.

## Conclusions

*Brinp1*^−/−^ mice exhibit reduced sociability, vocalisation impairments, hyperactivity and alterations in short-term memory. These behavioural phenotypes appear to show face validity for the core social communication deficits of ASD, along with the hyperactivity aspect of ADHD. The reported increase in PV-positive interneurons in the *Brinp1*^−/−^ mouse neocortex and hippocampus may underpin such behavioural changes. These findings demonstrate an important role in normal brain development and suggest *Brinp1* as a candidate gene for neurodevelopmental disorders, with closest relevance to ASD.

## Additional files

Additional file 1: Figure S1. Additional behavioural tests. **a** Normal vision of *Brinp1* ^−/−^ mice when lowered onto a wire mesh; female: *t*(10) = 0.7670, *p* = 0.4608, male: *t*(10) = 0.00, *p* > 0.99. *N* = 6 females, 6 male mice per genotype. **b** Normal olfaction of *Brinp1* ^−/−^ mice, tested as exploration time of an attractive olfaction (peanut butter) at dilutions of 1 × 10^−1^, 1 × 10^−2^ and 1 × 10^−3^; female: *F*(1,10) = 0.362, *p* = 0.561, male: *F*(1,10) = 1.163, *p* = 0.306, repeat measures two-way ANOVA. Water was used as a control. *N* = 6 female, 6 male mice per genotype. **c** *Brinp1* ^−/−^ mice did not show significant motor co-ordination impairment on the Rotarod; female: *F*(1,20) = 0.175, *p* = 0.680, male: *F*(1,20) = 2.189, *p* = 0.155, repeat measures two-way ANOVA. Results combined from two cohorts tested. *N* = 11 female, 11 male mice per genotype. Data presented as the mean ± SE. **d** Elevated plus maze, combined data for male and female mice: (i) *Brinp1* ^*−/−*^ mice spent significantly less time in the closed arms of the EPM *t*(22) = 2.538, *p* = 0.0188, unpaired Student’s *t* test, *n* = 12 mice per genotype. (ii) No significant difference for number of entries into either the open or closed arm of the EPM. **e** Male *Brinp1* ^−/−^ mice are normal for the novel object recognition test (NORT). Recognition of novelty represented by a recognition index, comparing percentage time investigating the novel object. Experiment was carried out using methods described previously [47], with a 3 min training/10 min retention session. *N* = 8 WT and 7 *Brinp1* ^−/−^ mice. **f** WT and *Brinp1* ^−/−^ mice locomotor activity following injection of MPH/saline in the locomotor cell. An acute IP injection of 1.25 mg/kg of MPH increased locomotor activity for both WT and *Brinp1* ^−/−^ mice, *N* = 10 WT MPH, 10 *Brinp1* ^−/−^ MPH, 10 WT saline, 10 *Brinp1* ^−/−^ saline. Repeat measures ANOVA analysis performed on distance travelled post injection (20 min+) revealed an effect of genotype; female: *F*(1,16) = 15.882, *p* < 0.001, male: *F*(1,16) = 10.317, *p* = 0.005. Whist a trend of increased activity was evident after administration of 1.25 mg/kg of MPH, the overall effect of the drug on distance travelled was non-significant at this lower dose; female: *F*(1,16) = 1.987, *p* = 0.178, male *F*(1,16) = 2.537, *p* = 0.131. **p* < 0.05, ***p* < 0.01, ****p* < 0.001. Data represented as the mean ± SD except where otherwise stated. (PNG 327 kb) Additional file 2: Figure S2. Immunohistochemistry of adult Brinp1 knock-out mice brains. **a** No significant difference in number of calretinin (CR)-positive cells in the somatosensory (SS) neocortex of *Brinp1* ^−/−^ mice, *t*(6) = 0.9625, *p* = 0.3730, unpaired Student’s *t* test. **b** No significant difference in number of somatostatin (SST)-positive cells in the somatosensory (SS) neocortex of *Brinp1* ^−/−^ mice, *t*(6) = 0.5406, *p* = 0.6082, unpaired Student’s *t* test. **c** No significant change in GFAP+ cells in the somatosensory (SS) cortex of *Brinp1* ^−/−^ mice, *t*(6) = 0.8765, *p* = 0.4209, unpaired Student’s *t* test. **d** No significant change in GFAP+ cells in the somatosensory hippocampus of *Brinp1* ^−/−^ mice, *t*(6) = 0.3222, *p* = 0.7567, unpaired Student’s *t* test. *N* = 4 WT, 4 *Brinp1* ^−/−^ mice. **p* < 0.05, ***p* < 0.01. All data represented as the mean ± SD. (PNG 1999 kb) Additional file 3: Figure S3. Pyramidal neuron distribution in the adult *Brinp1* ^−/−^ somatosensory neocortex. **a** Normal density of NeuN+ cells in the *Brinp1* ^−/−^ somatosensory neocortex. **b** No significant change in distribution of NeuN+ cells through somatosensory cortical layers, *F*(1,6) = 1.423, *p* = 0.278, repeat measures two-way ANOVA. Sections counterstained with DAPI. **c **No significant changes in Cux1 cell number or distribution were detected in the *Brinp1* ^−/−^ somatosensory neocortex, *F*(1,6) = 2.027, *p* = 0.204, repeat measures two-way ANOVA. *N* = 4 WT, 4 *Brinp1* ^−/−^ mice. **p* < 0.05, ***p* < 0.01. All data represented as the mean ± SD. (PNG 2569 kb) Additional file 4: Figure S4. Up-regulation of Astrotactin 1 and Astrotactin 2 mRNA in the embryonic brain and adult hippocampus of *Brinp1* knock-out mice (normalised with actin). **a** qPCR showing a significant increase in *Astn2* mRNA, *t*(9) = 2.829, *p* = 0.0222, in the developing (E18.5) mouse brain. Levels of exon 3-deleted *Brinp1* mRNA (*Brinp1*Δe3) also increase *t*(9) = 2.733, *p* = 0.0231, unpaired Student’s *t* tests. No significant changes overserved for *Brinp2* mRNA levels or *Brinp3* mRNA levels. **b** An increase in *Astn1* and *Astn2* expression was also detectable in the hippocampus at 6 weeks of the *Brinp1* ^−/−^ mice: *Astn1*: *t*(9) = 3.384, *p* = 0.0081, *Astn2*: *t*(9) = 2.821, *p* = 0.0200, unpaired Student’s *t* tests. No significant changes were detected in levels of *Brinp1*Δe3 mRNA, *Brinp2* mRNA or *Brinp3* mRNA. **c** No significant change in expression of *Brinps or* Astrotactins in the 6-week-old *Brinp1* ^−/−^ cortex. *N* = 3 WT, 4 *Brinp1* ^−/−^, **p* < 0.05, ***p* < 0.01. Normalisation against actin expression levels. All data represented as the mean ± SE. (PNG 70 kb)

## Abbreviations

ADHD: attention deficit hyperactivity disorder
ASD: autism spectrum disorder
ANOVA: analysis of variance
ASTN: astrotactin
BAC: bacterial artificial chromosome
BRINP or DBC: bone morphogenetic protein/retinoic acid-inducible neural-specific protein
BrdU: bromodeoxyuridine
CNV: copy number variation
DAPI: 4′,6-diamidino-2-phenylindole
FRT: flippase (Flp) recognition target
GAPDH: glyceraldehyde-3-phosphate dehydrogenase
GFAP: glial fibrillary acidic protein
GWAS: genome-wide association study
IP: intraperitoneal
MACPF: Membrane Attack Complex/Perforin
MPH: methylphenidate
MWM: Morris water maze
NDD: neurodevelopmental disorder
PBS: phosphate-buffered saline
PFA: paraformaldehyde
PPI: pre-pulse inhibition
PV: parvalbumin
SNP: single nucleotide polymorphisms
USV: ultrasonic vocalisation

## References

[1] Iossifov I (2014). The contribution of de novo coding mutations to autism spectrum disorder. Nature.

[2] Sebat J (2007). Strong association of de novo copy number mutations with autism. Science.

[3] Weiss LA (2009). Autism genetics: emerging data from genome-wide copy-number and single nucleotide polymorphism scans. Expert Rev Mol Diagn.

[4] Folstein SE, Rosen-Sheidley B (2001). Genetics of autism: complex aetiology for a heterogeneous disorder. Nat Rev Genet.

[5] Fombonne E (2005). Epidemiology of autistic disorder and other pervasive developmental disorders. J Clin Psychiatry.

[6] Brugha TS (2011). Epidemiology of autism spectrum disorders in adults in the community in England. Arch Gen Psychiatry.

[7] First, M.B. and APA, *DSM-5 handbook of differential diagnosis*. First edition. ed. 2013. xv, 322 pages.

[8] Tek S (2014). Longitudinal analyses of expressive language development reveal two distinct language profiles among young children with autism spectrum disorders. J Autism Dev Disord.

[9] McGonigle-Chalmers M (2013). Profound expressive language impairment in low functioning children with autism: an investigation of syntactic awareness using a computerised learning task. J Autism Dev Disord.

[10] Rybakowski F (2014). Autism spectrum disorders—epidemiology, symptoms, comorbidity and diagnosis. Psychiatr Pol.

[11] Tureck K (2014). An examination of the relationship between autism spectrum disorder, intellectual functioning, and comorbid symptoms in children. Res Dev Disabil.

[12] Simonoff E (2008). Psychiatric disorders in children with autism spectrum disorders: prevalence, comorbidity, and associated factors in a population-derived sample. J Am Acad Child Adolesc Psychiatry.

[13] Wohr M (2015). Lack of parvalbumin in mice leads to behavioral deficits relevant to all human autism core symptoms and related neural morphofunctional abnormalities. Transl Psychiatry.

[14] Lawrence YA (2010). Parvalbumin-, calbindin-, and calretinin-immunoreactive hippocampal interneuron density in autism. Acta Neurol Scand.

[15] Vogt D (2015). The parvalbumin/somatostatin ratio is increased in Pten mutant mice and by human PTEN ASD alleles. Cell Rep.

[16] Rossignol E (2011). Genetics and function of neocortical GABAergic interneurons in neurodevelopmental disorders. Neural Plast.

[17] Marin O (2012). Interneuron dysfunction in psychiatric disorders. Nat Rev Neurosci.

[18] Rosado CJ (2008). The MACPF/CDC family of pore-forming toxins. Cell Microbiol.

[19] Kondos SC (2010). The structure and function of mammalian membrane-attack complex/perforin-like proteins. Tissue Antigens.

[20] Kawano H (2004). Identification and characterization of novel developmentally regulated neural-specific proteins, BRINP family. Brain Res Mol Brain Res.

[21] Terashima M (2010). Analysis of the expression and function of BRINP family genes during neuronal differentiation in mouse embryonic stem cell-derived neural stem cells. J Neurosci Res.

[22] Kobayashi M (2014). Absence of BRINP1 in mice causes increase of hippocampal neurogenesis and behavioral alterations relevant to human psychiatric disorders. Mol Brain.

[23] Wood WE (2008). Dietary retinoic acid affects song maturation and gene expression in the song system of the zebra finch. Dev Neurobiol.

[24] Lovell PV, et al. Birdsong “Transcriptomics”: Neurochemical Specializations of the Oscine Song System. 2008 10.1371/journal.pone.0003440 PMC2563692 18941504

[25] Beetz C (2005). Low expression but infrequent genomic loss of the putative tumour suppressor DBCCR1 in astrocytoma. Oncol Rep.

[26] Gao S (2004). Loss of heterozygosity at 9q33 and hypermethylation of the DBCCR1 gene in oral squamous cell carcinoma. Br J Cancer.

[27] Nishiyama H, et al. Negative regulation of G1S transition by the candidate bladder tumour supressor gene DBCCR1. 2001. 10.1038/sj.onc.1204432 11420708

[28] Adams NC, et al. Mice that lack astrotactin have slowed neuronal migration. Development, 2002 10.1242/dev.129.4.965 11861479

[29] Wilson PM (2010). Astn2, a novel member of the astrotactin gene family, regulates the trafficking of ASTN1 during glial-guided neuronal migration. J Neurosci.

[30] Vawter MP, Mamdani F, Macciardi F (2011). An integrative functional genomics approach for discovering biomarkers in schizophrenia. Brief Funct Genomics.

[31] Do C, et al. Web-based genome-wide association study identifies two novel loci and a substantial genetic component for Parkinson’s Disease. 2011 10.1371/journal.pgen.1002141 PMC3121750 21738487

[32] Edwards TL (2010). Genome-wide association study confirms SNPs in SNCA and the MAPT region as common risk factors for Parkinson disease. Ann Hum Genet.

[33] Heinzen EL (2010). Genome-wide scan of copy number variation in late-onset Alzheimer’s disease. J Alzheimers Dis.

[34] Glessner JT (2009). Autism genome-wide copy number variation reveals ubiquitin and neuronal genes. Nature.

[35] Lionel AC (2014). Disruption of the ASTN2/TRIM32 locus at 9q33.1 is a risk factor in males for autism spectrum disorders, ADHD and other neurodevelopmental phenotypes. Hum Mol Genet.

[36] Lionel AC (2011). Rare copy number variation discovery and cross-disorder comparisons identify risk genes for ADHD. Sci Transl Med.

[37] Vrijenhoek T (2008). Recurrent CNVs disrupt three candidate genes in schizophrenia patients. Am J Hum Genet.

[38] Teoh SS (2014). Maspin is not required for embryonic development or tumour suppression. Nat Commun.

[39] Teoh SS (2012). A versatile monoclonal antibody specific to human SERPINB5. Hybridoma (Larchmt).

[40] Silverman JL (2010). Behavioural phenotyping assays for mouse models of autism. Nat Rev Neurosci.

[41] Maggio JC, Maggio JH, Whitney G (1983). Experience-based vocalization of male mice to female chemosignals. Physiol Behav.

[42] Scattoni ML (2008). Unusual repertoire of vocalizations in the BTBR T + tf/J mouse model of autism. PLoS One.

[43] Hiller MM (1996). ER degradation of a misfolded luminal protein by the cytosolic ubiquitin-proteasome pathway. Science.

[44] Wöhr M. Ultrasonic communication in mouse models of autism. Proceedings of Measuring Behavior 2012

[45] Kordon A (2011). Exploring the impact of once-daily OROS(R) methylphenidate (MPH) on symptoms and quality of life in children and adolescents with ADHD transitioning from immediate-release MPH. Postgrad Med.

[46] Huang FL, Huang KP (2012). Methylphenidate improves the behavioral and cognitive deficits of neurogranin knockout mice. Genes Brain Behav.

[47] Ishisaka M (2012). Diacylglycerol kinase beta knockout mice exhibit attention-deficit behavior and an abnormal response on methylphenidate-induced hyperactivity. PLoS One.

[48] Hess EJ, Collins CA, Wilson MC (1996). Mouse model of hyperkinesis implicates SNAP-25 in behavioural regulation. J Neurosci.

[49] Scattoni ML, Crawley J, Ricceri L (2009). Ultrasonic vocalizations: a tool for behavioural phenotyping of mouse models of neurodevelopmental disorders. Neurosci Biobehav Rev.

[50] Jamain S (2008). Reduced social interaction and ultrasonic communication in a mouse model of monogenic heritable autism. Proc Natl Acad Sci U S A.

[51] Yang M (2012). Reduced excitatory neurotransmission and mild autism-relevant phenotypes in adolescent Shank3 null mutant mice. J Neurosci.

[52] Chien SC (2010). A new familial insertion, ins(18;9)(q12.2;q33.1q31.1) with a 9q31.1-9q33.1 deletion in a girl with a cleft lip and palate. Am J Med Genet A.

[53] van der Meere J (1995). Sustained attention, activation and MPH in ADHD: a research note. J Child Psychol Psychiatry.

[54] Contini V (2013). Pharmacogenetics of response to methylphenidate in adult patients with attention-deficit/hyperactivity disorder (ADHD): a systematic review. Eur Neuropsychopharmacol.

[55] Giros B (1996). Hyperlocomotion and indifference to cocaine and amphetamine in mice lacking the dopamine transporter. Nature.

[56] Bymaster FP (2002). Atomoxetine increases extracellular levels of norepinephrine and dopamine in prefrontal cortex of rat: a potential mechanism for efficacy in attention deficit/hyperactivity disorder. Neuropsychopharmacology.

[57] Hunt RD, Arnsten AF, Asbell MD (1995). An open trial of guanfacine in the treatment of attention-deficit hyperactivity disorder. J Am Acad Child Adolesc Psychiatry.

[58] Lovett-Barron M (2012). Regulation of neuronal input transformations by tunable dendritic inhibition. Nat Neurosci.

[59] Enticott PG (2013). GABAergic activity in autism spectrum disorders: an investigation of cortical inhibition via transcranial magnetic stimulation. Neuropharmacology.

[60] Thatcher RW (2009). Autism and EEG phase reset: deficient GABA mediated inhibition in thalamo-cortical circuits. Dev Neuropsychol.

[61] Rubenstein JL, Merzenich MM (2003). Model of autism: increased ratio of excitation/inhibition in key neural systems. Genes Brain Behav.

[62] Scacheri PC (2001). Bidirectional transcriptional activity of PGK-neomycin and unexpected embryonic lethality in heterozygote chimeric knockout mice. Genesis.

[63] Pham CT (1996). Long-range disruption of gene expression by a selectable marker cassette. Proc Natl Acad Sci U S A.

