# Supplementary figures and images for: Brinp1−/− mice exhibit autism-like behaviour, altered memory, hyperactivity and increased parvalbumin-positive cortical interneuron density
Source: Mol Autism. 2016 Mar 31;7:22. doi: 10.1186/s13229-016-0079-7 (PMC4818446; doi:10.1186/s13229-016-0079-7)

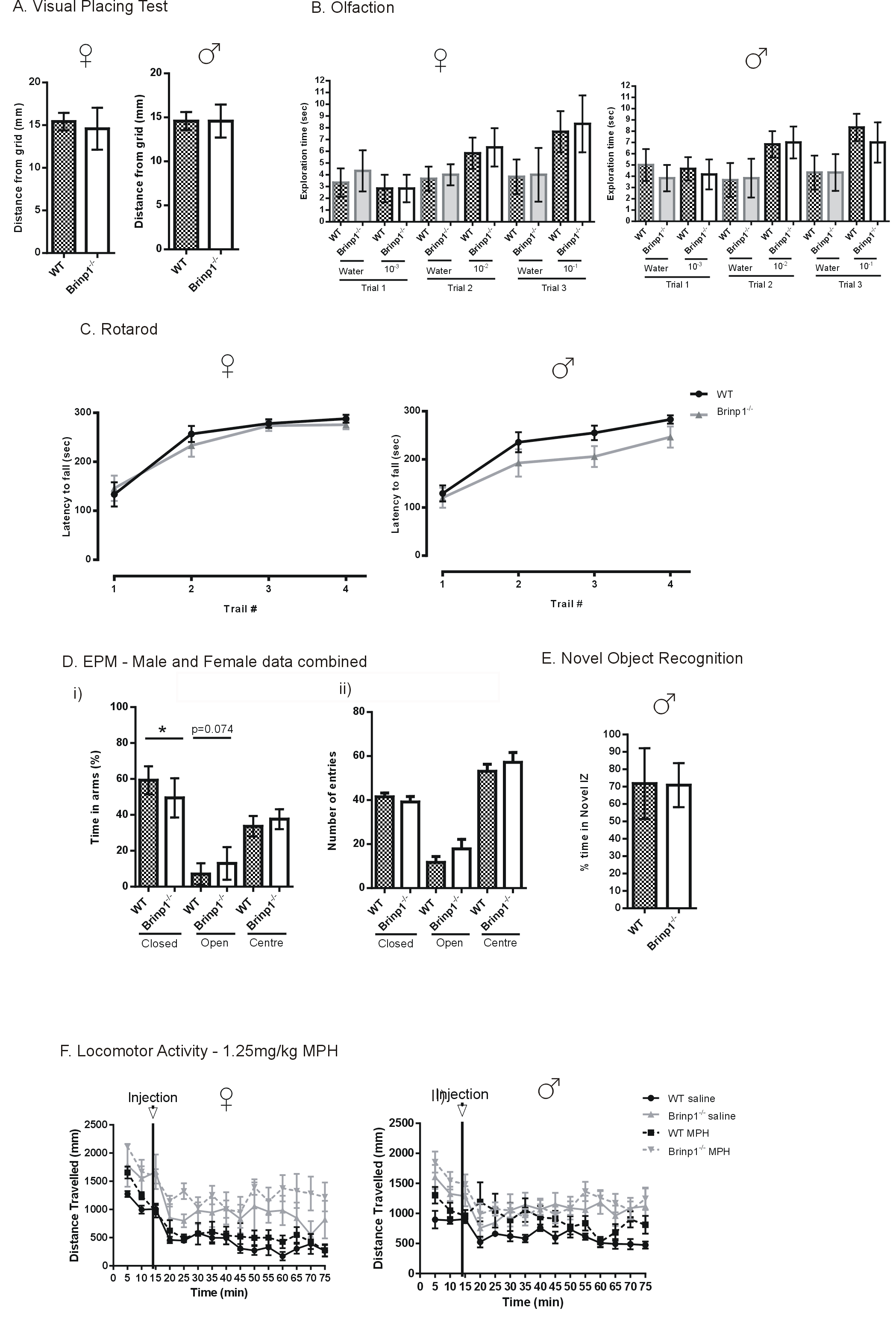

Supplement: Additional file 1: Figure S1. — Additional behavioural tests. a Normal vision of Brinp1 −/− mice when lowered onto a wire mesh; female: t(10) = 0.7670, p = 0.4608, male: t(10) = 0.00, p > 0.99. N = 6 females, 6 male mice per genotype. b Normal olfaction of Brinp1 −/− mice, tested as exploration time of an attractive olfaction (peanut butter) at dilutions of 1 × 10^−1^, 1 × 10^−2^ and 1 × 10^−3^; female: F(1,10) = 0.362, p = 0.561, male: F(1,10) = 1.163, p = 0.306, repeat measures two-way ANOVA. Water was used as a control. N = 6 female, 6 male mice per genotype. c Brinp1 −/− mice did not show significant motor co-ordination impairment on the Rotarod; female: F(1,20) = 0.175, p = 0.680, male: F(1,20) = 2.189, p = 0.155, repeat measures two-way ANOVA. Results combined from two cohorts tested. N = 11 female, 11 male mice per genotype. Data presented as the mean ± SE. d Elevated plus maze, combined data for male and female mice: (i) Brinp1 −/− mice spent significantly less time in the closed arms of the EPM t(22) = 2.538, p = 0.0188, unpaired Student’s t test, n = 12 mice per genotype. (ii) No significant difference for number of entries into either the open or closed arm of the EPM. e Male Brinp1 −/− mice are normal for the novel object recognition test (NORT). Recognition of novelty represented by a recognition index, comparing percentage time investigating the novel object. Experiment was carried out using methods described previously [47], with a 3 min training/10 min retention session. N = 8 WT and 7 Brinp1 −/− mice. f WT and Brinp1 −/− mice locomotor activity following injection of MPH/saline in the locomotor cell. An acute IP injection of 1.25 mg/kg of MPH increased locomotor activity for both WT and Brinp1 −/− mice, N = 10 WT MPH, 10 Brinp1 −/− MPH, 10 WT saline, 10 Brinp1 −/− saline. Repeat measures ANOVA analysis performed on distance travelled post injection (20 min+) revealed an effect of genotype; female: F(1,16) = 15.882, p < 0.001, male: F(1,16) = 10.317, p = 0.005. Whist a trend of increased [file 13229_2016_79_MOESM1_ESM.png]

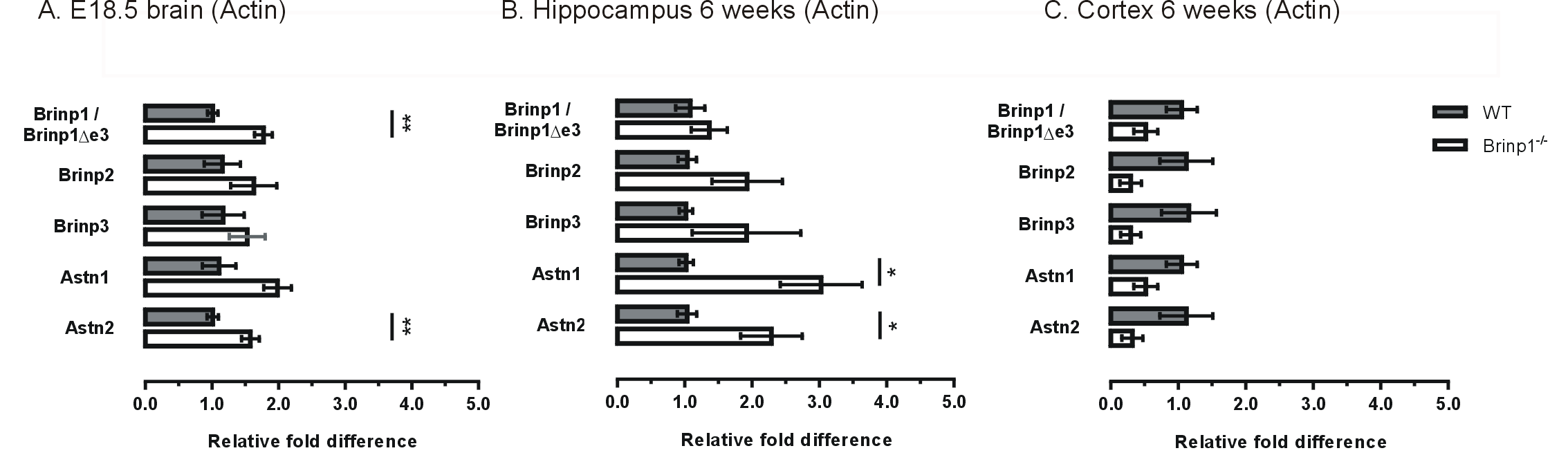

Supplement: Additional file 2: Figure S2. — Immunohistochemistry of adult Brinp1 knock-out mice brains. a No significant difference in number of calretinin (CR)-positive cells in the somatosensory (SS) neocortex of Brinp1 −/− mice, t(6) = 0.9625, p = 0.3730, unpaired Student’s t test. b No significant difference in number of somatostatin (SST)-positive cells in the somatosensory (SS) neocortex of Brinp1 −/− mice, t(6) = 0.5406, p = 0.6082, unpaired Student’s t test. c No significant change in GFAP+ cells in the somatosensory (SS) cortex of Brinp1 −/− mice, t(6) = 0.8765, p = 0.4209, unpaired Student’s t test. d No significant change in GFAP+ cells in the somatosensory hippocampus of Brinp1 −/− mice, t(6) = 0.3222, p = 0.7567, unpaired Student’s t test. N = 4 WT, 4 Brinp1 −/− mice. *p < 0.05, **p < 0.01. All data represented as the mean ± SD. (PNG 1999 kb) [file 13229_2016_79_MOESM2_ESM.png]

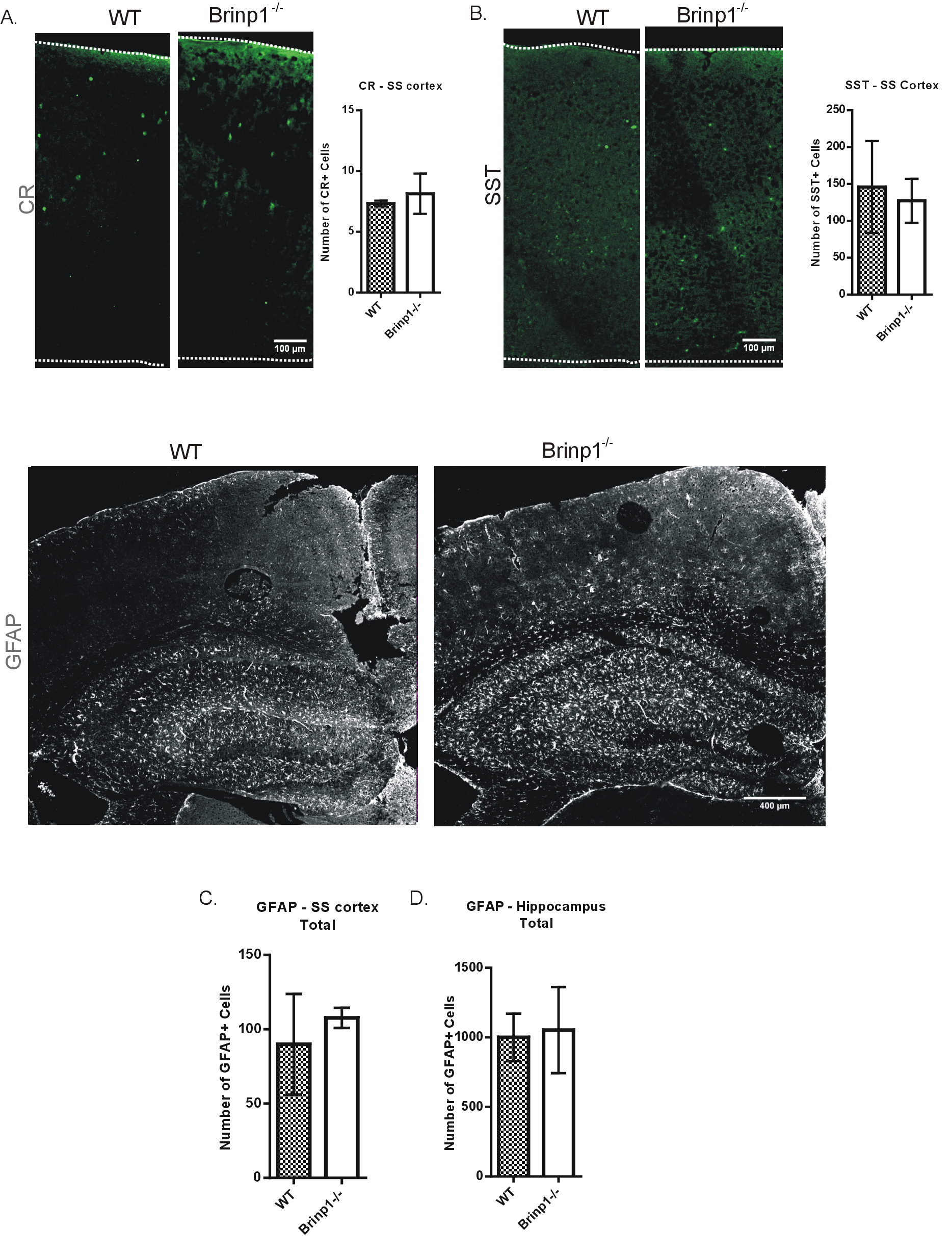

Supplement: Additional file 3: Figure S3. — Pyramidal neuron distribution in the adult Brinp1 −/− somatosensory neocortex. a Normal density of NeuN+ cells in the Brinp1 −/− somatosensory neocortex. b No significant change in distribution of NeuN+ cells through somatosensory cortical layers, F(1,6) = 1.423, p = 0.278, repeat measures two-way ANOVA. Sections counterstained with DAPI. c No significant changes in Cux1 cell number or distribution were detected in the Brinp1 −/− somatosensory neocortex, F(1,6) = 2.027, p = 0.204, repeat measures two-way ANOVA. N = 4 WT, 4 Brinp1 −/− mice. *p < 0.05, **p < 0.01. All data represented as the mean ± SD. (PNG 2569 kb) [file 13229_2016_79_MOESM3_ESM.png]

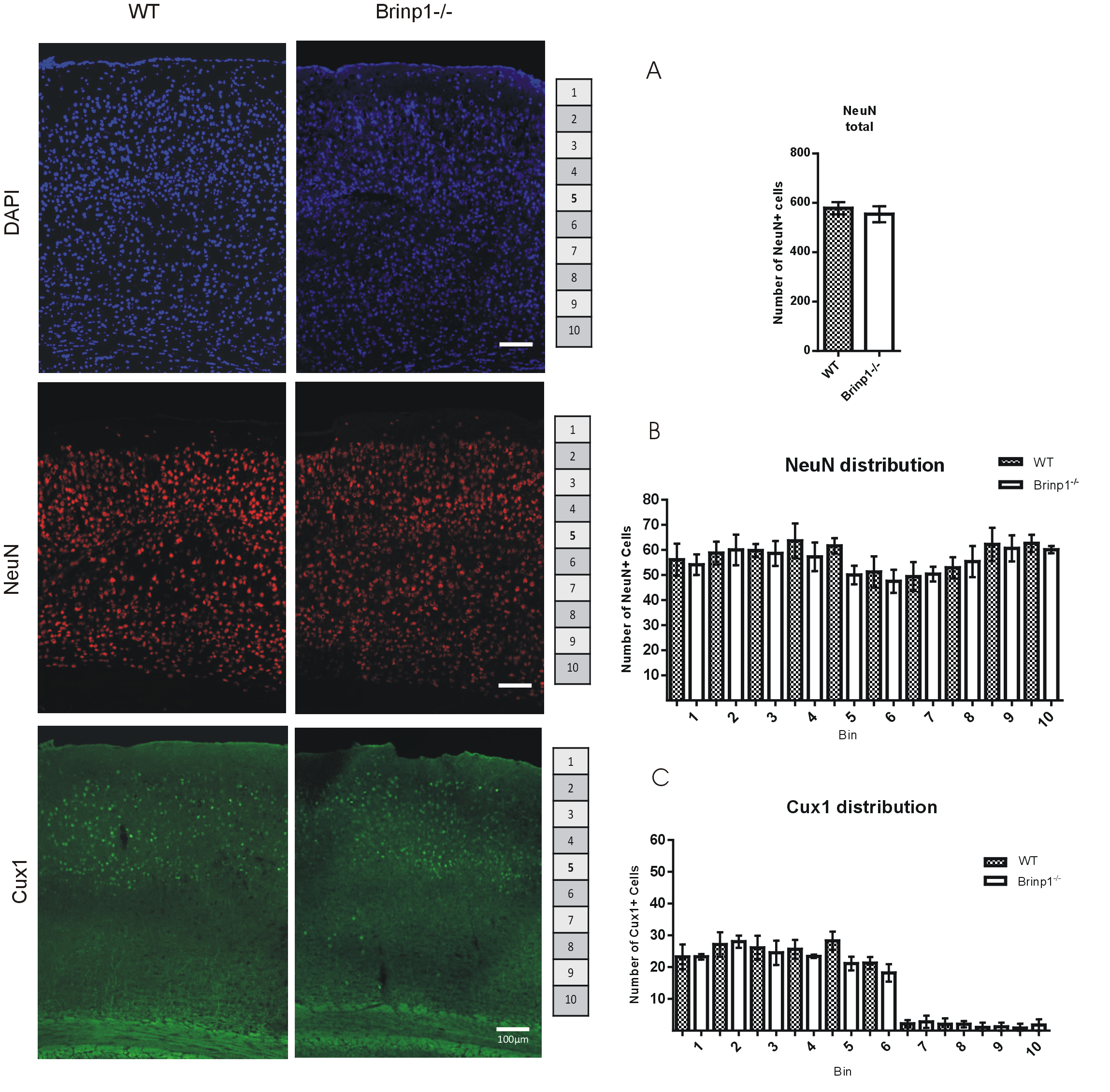

Supplement: Additional file 4: Figure S4. — Up-regulation of Astrotactin 1 and Astrotactin 2 mRNA in the embryonic brain and adult hippocampus of Brinp1 knock-out mice (normalised with actin). a qPCR showing a significant increase in Astn2 mRNA, t(9) = 2.829, p = 0.0222, in the developing (E18.5) mouse brain. Levels of exon 3-deleted Brinp1 mRNA (Brinp1Δe3) also increase t(9) = 2.733, p = 0.0231, unpaired Student’s t tests. No significant changes overserved for Brinp2 mRNA levels or Brinp3 mRNA levels. b An increase in Astn1 and Astn2 expression was also detectable in the hippocampus at 6 weeks of the Brinp1 −/− mice: Astn1: t(9) = 3.384, p = 0.0081, Astn2: t(9) = 2.821, p = 0.0200, unpaired Student’s t tests. No significant changes were detected in levels of Brinp1Δe3 mRNA, Brinp2 mRNA or Brinp3 mRNA. c No significant change in expression of Brinps or Astrotactins in the 6-week-old Brinp1 −/− cortex. N = 3 WT, 4 Brinp1 −/−, *p < 0.05, **p < 0.01. Normalisation against actin expression levels. All data represented as the mean ± SE. (PNG 70 kb) [file 13229_2016_79_MOESM4_ESM.png]
